# The *Verticillium dahliae* SnodProt1-Like Protein VdCP1 Contributes to Virulence and Triggers the Plant Immune System

**DOI:** 10.3389/fpls.2017.01880

**Published:** 2017-10-31

**Authors:** Yi Zhang, Yuhan Gao, Yingbo Liang, Yijie Dong, Xiufen Yang, Jingjing Yuan, Dewen Qiu

**Affiliations:** Institute of Plant Protection (CAAS), Haidian, China

**Keywords:** *Verticillium dahlia*, cerato-platanin, SnodProt1, virulence, elicitor, plant immunity

## Abstract

During pathogenic infection, hundreds of proteins that play vital roles in the *Verticillium dahliae*-host interaction are secreted. In this study, an integrated proteomic analysis of secreted *V*. *dahliae* proteins was performed, and a conserved secretory protein, designated VdCP1, was identified as a member of the SnodProt1 phytotoxin family. An expression analysis of the *vdcp1* gene revealed that the transcript is present in every condition studied and displays elevated expression throughout the infection process. To investigate the natural role of VdCP1 in *V. dahliae*, two *vdcp1* knockout mutants and their complementation strains were generated. Bioassays of these mutants revealed no obvious phenotypic differences from the wild-type (WT) in terms of mycelial growth, conidial production or mycelial/spore morphology. However, compared with the WT, the *vdcp1* knockout mutants displayed attenuated pathogenicity in cotton plants. Furthermore, treating plants with purified recombinant VdCP1 protein expressed in *Pichia pastoris* induced the accumulation of reactive oxygen species (ROS), expression of several defense-related genes, leakage of ion electrolytes, enhancement of defense-related enzyme activity and production of salicylic acid. Moreover, VdCP1 conferred resistance to *Botrytis cinerea* and *Pseudomonas syringae* pv. tabaci in tobacco and to *V. dahliae* in cotton. Further research revealed that VdCP1 possesses chitin-binding properties and that the growth of *vdcp1* knockout mutants was more affected by treatments with chitinase, indicating that VdCP1 could protect *V. dahliae* cell wall from enzymatic degradation, which suggests an effector role of VdCP1 in infecting hosts.

## Introduction

*Verticillium dahliae* is a soil-borne plant pathogen that causes *Verticillium* wilt, a destructive disease of cotton worldwide, and it also affects a wide range of dicotyledonous plants (Klosterman et al., 2009). Previous studies have demonstrated that proteins secreted by *V. dahliae*, including cell-wall-degrading enzymes (CWDEs) that degrade the plant cell wall for successful infection, and phytotoxins, which are toxic factors that lead to wilt symptoms, play key roles in the *V. dahliae*-plant interactions (Fradin and Thomma, 2006; Shi and Li, 2008). Additionally, some secretory proteins can induce cell death and/or elicit plant defense responses (Fradin and Thomma, 2006; Klosterman et al., 2009; Wang et al., 2011).

SnodProt1 was identified from the phytotoxic wheat pathogen *Stagonospora nodorum* (Skinner et al., 2001). It is a member of the cerato-platanin protein (CPP) family (Pfam family PF07249), which was first isolated from the phytotoxic tree pathogen *Ceratocystis fimbriata* f. sp. platani (Pazzagli et al., 1999). CPPs possess four conserved cysteines, low molecular weights and high hydrophobicity and are conserved in filamentous fungi. It has been shown that CPPs can function as elicitors and/or virulence factors in hosts. Many CPPs that trigger plant immunity have been identified to date, including Epl1 (eliciting plant response-like protein 1) from *Trichoderma atroviride* (Seidl et al., 2006), Sm1 (small protein 1) from *Trichoderma virens* (Djonovic et al., 2006) and BcSpl1 from *Botrytis cinerea* (Frías et al., 2011), while several CPPs, including BcSpl1, MSP1 from *Magnaporthe grisea* (Jeong et al., 2007) and CP from *Ceratocystis fimbriata* (Scala et al., 2004), have been shown to act as virulence factors. However, no SnodProt1-like proteins have been characterized in *V. dahliae*.

Although efforts have been made to decipher the biological functions of CPPs, their roles in fungus-plant interactions are unclear. CPPs are abundantly secreted into the culture medium (Seidl et al., 2006; González-Fernández et al., 2014), but primarily bind to the fungal cell wall (Boddi, 2004; Frías et al., 2013), and exhibit chitin-binding and expansin-like properties (Baccelli et al., 2013; de O Barsottini et al., 2013; Frischmann et al., 2013). Chitin is an important structural component of the fungal cell wall. And expansin-like proteins have cell wall-loosening activity and have various roles in growth and developmental processes. These properties suggest that CPPs may play important roles in fungal cell wall expansion and in chitin oligomer scavenging, thereby disguising fungal presence and preventing the recognition of fungi by plants (Frischmann et al., 2013; Gaderer et al., 2014). The specific mechanism by which CPPs contribute to virulence, however, remains undefined.

BcSpl1 has been shown to be perceived by the plant immune system as pathogen-associated molecular pattern (PAMP) (Frías et al., 2011). PAMPs are recognized by pattern recognition receptors (PRRs) located in the plasma membrane and trigger a primary plant immune response known as PAMP-triggered immunity (PTI). PTI provides plants with basal resistance against pathogen invasion and plays a vital role in maintaining resistance. The generation of reactive oxygen species (ROS) is an early defense response in PTI that generally plays a significant role in downstream signal transduction and is associated with the hypersensitive response (HR), a type of programmed cell death (Frías et al., 2011). The HR further leads to leaf necrosis and defense gene activation (Frías et al., 2011; Mengiste, 2012). BcSpl1 and CP have strong necrosis-inducing activity in infiltrated plant leaves (Pazzagli et al., 1999; Frías et al., 2011). However, the Epl1, Sm1 and MSP1 do not cause necrosis, although they can induce plant defense responses such as ROS production and defense-related gene expression (Djonovic et al., 2006; Seidl et al., 2006; Jeong et al., 2007). The necrosis-inducing activity of CPPs is ambiguous.

In the present study, we first characterized a member of the SnodProt1 family, VdCP1, which was identified in the *V. dahliae* secretome. Purified recombinant VdCP1 protein induced local and systemic defense responses in host plants and conferred plants disease resistance against pathogens. Furthermore, the pathogenicity of *vdcp1* knockout mutants was impaired compared with that of the wild-type (WT). Our results demonstrate that VdCP1 contributes to virulence in *V. dahliae*, triggers the plant immune system, and protects the *V. dahliae* cell wall from enzymatic degradation. This information provides new insight into the *V. dahliae*-cotton interactions.

## Materials and methods

### Plants and strains

Tobacco (*Nicotiana tabacum* cv. Samsun NN) and tomato (*Lycopersicon esculentum* cv. Zhongza-9) plants were grown in a greenhouse at 25°C, with a day/night period of 16/8 h, and cotton (*G. hirsutum* cv. Guoxin) plants were maintained at 28°C, with a day/night period of 16/8 h. *V. dahliae* strain XH-8 was preserved at the China General Microbiological Culture Collection of China (CGMCC no. 7611). *B. cinerea* strain BC-98 was preserved at the CGMCC (CGMCC no. 7057). The fungi were grown on potato dextrose agar medium (PDA) at 25°C in the dark. *Pseudomonas syringae* pv. tabaci and *Agrobacterium tumefaciens* AGL-1 were stored in 20% glycerol at −80°C and grown on the KB (Rif^50^) and LB (K^50^ and Rif^50^) medium plate at 28°C, respectively.

### Secretome of *V. dahliae*

1 L of CDB liquid medium (NaNO_3_, 3.0 g/L, MgSO_4_·7H_2_O, 0.5 g/L, KCl, 0.5 g/L, FeSO_4_·7H_2_O, 0.01 g/L, K_2_HPO_4_, 1.0 g/L, sucrose 30 g/L) with 5% roots fragments (w/v) from 6-week-old cotton plants was inoculated with 1 ml of a *V. dahliae* spore suspension (10^5^/ml) and subsequently cultured for 2 days in the dark at 25°C with shaking at 180 rpm. The culture filtrate was then centrifuged at 2,500 g for 30 min at 4°C, after which the supernatant was collected and passed through a 0.22 μm filter (Millipore, Suzhou, China). The proteins in the supernatant were precipitated with TCA as described in Wang et al. (2007). Protein concentrations were determined using a BCA™ Protein Assay Kit (TransGen Biotech, Beijing, China) with BSA as a standard. The mixture of proteins was analyzed via LC-MS/MS (Q Exactive, Bruker Daltonics K.K., Tokyo, Japan), combined with a BLAST search of the *V. dahliae* VdLs.17 genome database (http://www.broadinstitute.org/annotation/genome/verticillium_dahliae/MultiHome.html).

### Cloning, expression and purification of *vdcp1*

A 360 bp *vdcp1* fragment (amplified with primers *vdcp1*F/*vdcp1*R, Table S1) without the predicted signal peptides and stop codons was inserted into the pPICZαA plasmid at the *EcoR*I and *Xba*I sites. The recombinant plasmid pPICZαA-*vdcp1*, pPICZαA-*His*-*tag* or empty plasmid pPICZαA were linearized with *Pme*I and transformed into *Pichia pastoris* KM71H for expression. The transformants were cultured in BMGY (1% yeast extract, 2% peptone, 100 mM potassium phosphate, pH 6.0, 1.34% yeast nitrogen base, 4 × 10^−5^ % biotin, 1% glycerol) to culture OD_600_ = 6, after which the cell pellet was collected and resuspended in BMM (100 mM potassium phosphate, pH 6.0, 1.34% yeast nitrogen base, 4 × 10^−5^ % biotin, 0.5% methanol), followed by induction for 3 days with 0.5% methanol. The resulting culture supernatant was used for the purification of VdCP1 or His-tag (Zhang et al., 2017a,b). The purification was carried out with HisTrap HP pre-packed minicolumns (GE Healthcare Life Sciences, Uppsala, Sweden). The purified VdCP1 or His-tag was kept in protein buffer (20 mM Tris, pH 8.0). VdCP1 was analyzed via Tris-SDS-PAGE and confirmed using Western blotting for His-tag and with MS, and His-tag peptide was analyzed via Tris-Tricine-SDS-PAGE with the low molecular mass marker (RTD6110, Beijing Real-Times Biotechnology Co. Ltd., China) and confirmed by Western blotting. The concentration of the purified protein was measured using the BCA™ Protein Assay Kit, and the protein was then frozen at −80°C in small aliquots until use.

### Assays of defense responses

The necrosis-inducing activity, the production of ROS, and the measurement of ion electrolyte leakage, phenylalanine ammonia-lyase (PAL), polyphenol oxidase (PPO) and peroxidase (POD) activities and SA production were assayed in VdCP1-infiltrated plants. His-tag/BSA-infiltrated plants were used as controls. VdCP1, His-tag or BSA was infiltrated into one leaf of 4-week-old tobacco, cotton and tomato plants using a blunt 1-ml syringe, 24 h later the leaves were cut and treated with DAB (1 mg/ml, pH 3.8). Leaves were vacuum infiltrated and immersed with DAB solution for 3 h. To visualize DAB deposits, leaves were then incubated in absolute ethanol to eliminate chlorophyll and photographed (Frías et al., 2011). The DAB staining intensity on photographs was quantified using software ImageJ and is shown as the number of DAB pixels relative to total pixels according to a previous study (Chang et al., 2014). To assay electrolyte leakage, one leaf of 4-week-old tobacco plant was infiltrated with VdCP1, His-tag or BSA, three leaf discs of 1 cm diameter were cut at 6 hpi and submerged in 1 ml water at 4°C with shaking at 200 rpm, and the conductivity was measured at the indicated time points with a MP526 ion conductivity meter (LabSen, Shanghai, China) (Frías et al., 2011). Three tobacco plants were treated to detect ROS and electrolyte leakage in every time point, three biological replicates were carried out. One cotyledon of 4-week-old cotton plant was infiltrated with VdCP1, His-tag or BSA, and the leaves were collected to measure the activities of PAL, PPO and POD according to previously described methods (Hano et al., 2008; Zhang et al., 2010). 3 cotton plants were treated to measure the enzymes activities in every time point, three biological replicates were performed. At 72 h after infiltrating one of cotton cotyledons with VdCP1, His-tag or BSA, the roots were weighed and frozen in liquid nitrogen. For each sample, 0.1 g of the frozen tissue was extracted and quantitated for free SA as described previously (Lv et al., 2016). 3 cotton plants were treated and three biological replicates were performed.

### Characteristics of VdCP1

VdCP1 (30 μM) was incubated with 10 mg chitin from crab shells (catalog no. C9752, Sigma-Aldrich) in 50 mM sodium acetate buffer (1 ml, pH 6.5) at 38°C with shaking at 320 rpm (Baccelli et al., 2013; Frischmann et al., 2013). BSA at the same concentration was included as a control. After the indicated times, the tubes were centrifuged at 12,000 × g for 2 min, the supernatants were then transferred to new tubes, and the pellets were washed with buffer at least 3 times to remove any remaining protein solution. Next, the pellet was resuspended in buffer containing 2% SDS and incubated at 99°C. After 10 min, the suspension was centrifuged at 12,000 × g for 2 min, and the supernatant was transferred to a new tube and precipitated using the methanol and chloroform method (Wang et al., 2007). Unbound proteins from the supernatant and bound proteins from the pellet were analyzed via sodium dodecyl sulfate-polyacrylamide gel electrophoresis (SDS-PAGE). In addition, VdCP1 (30 μM) was incubated with 1 cm-diameter Whatman no. 1 filter paper (Whatman, Milan, Italy) in 50 mM sodium acetate buffer (1 ml, pH 6.5) (Baccelli et al., 2013). Protein buffer only or buffer containing BSA at the highest concentration were used as negative controls, and protein CP expressed in *P*. *pastoris* KM71H was used as positive control. At the end of the incubation period (48 h, 38°C, 320 rpm), the presence of paper fragments in the suspension was detected via spectrophotometric measurements of absorbance at 500 nm and photographed.

### Treatment of *V. dahliae* with chitinase

The wells of a microtitre plate were filled with 200 μl of potato dextrose broth (PDB) at pH 6.85 containing 0.2 U of chitinase from *Streptomyces griseus* (catalog no. C6137, Sigma-Aldrich) that had been resuspended in the buffer (50 mM potassium phosphate, pH 6.0). The controls were carried out by adding the buffer instead of the chitinase solutions. A final concentration of 10^6^/ml spores of *V. dahliae* WT, Δ*vdcp1* and Δ*vdcp1*-Res strains was added into PDB, and 68 μg/ml of resazurin dye (catalog no. R7017, Sigma-Aldrich) was added to each well to measure fungal growth. Resazurin is reduced from blue to light pink color in presence of actively growing cells. The plates were incubated at 25°C in the dark, and the absorbance was measured at 578 nm at the indicated times. *V. dahliae* growth inhibition. The percentage of *V. dahliae* growth inhibition was calculated as 100 minus the percentage of the ratio between net absorbance values of each sample and net absorbance of the medium with the resazurin dye but without *V. dahliae* (Sella et al., 2014; Quarantin et al., 2016).

### Generation of *vdcp1* deletion mutants and complementary mutants

*V. dahliae* with mutations in the *vdcp1* (Δ*vdcp1*) gene and *vdcp1* complementary strain (Δ*vdcp1*-Res) were constructed using the *A. tumefaciens*-mediated transformation method (Liu et al., 2013). Primers *vdcp1*aF/*vdcp1*aR, *vdcp1*bF/*vdcp1*bR and Hyg-F/Hyg-R were used to amplify the 1000-bp 5′ region of vdcp1, the 1000-bp 3′ region of vdcp1 and the 1800-bp hygromycin resistance cassette, respectively. A nested PCR reaction was carried out with primers Nest-F/Nest-R, which contain gateway BP reaction adaptors, these were then employed to amplify the fusion PCR sequence from the fusion of the three amplicons. Subsequently, a recombinant plasmid was constructed via homologous recombination between the final fusion amplicon and the pGKO2-Gateway vector, which contains the herpes simplex virus thymidine kinase (*HSVtk*) gene in T-DNA and was used as a lethal gene for counterselection against ectopic transformants with 5-fluoro-2′-deoxyuridine (F2dU). To generate the *vdcp1* complementation vector, the primers *vdcp1*CF and *vdcp1*CR, which contain *Kpn*I and *Xba*I enzyme sites, respectively, were employed to amplify the sequence containing *vdcp1* and its 1,100-bp 5′ and 3′ regions from the *V. dahliae* genome. The *vdcp1* cassette was ligated into the pCAM vector (Liu et al., 2013), which contains geneticin and hygromycin resistance selection markers. All primers are listed in Table S1. The *Agrobacterium* strain AGL-1 was used to transform the recombinant plasmid into *V. dahliae* spores.

### Quantitative real-time PCR

To analyse the expression of *vdcp1* gene, *V. dahliae* was cultured and collected from different culture media. The stem base of 2-week-old cotton plant was inoculated with a spore suspension of 10^8^/ml, and the inoculated tissues were collected at the indicated time points. 3 plants were inoculated for the assays in every time point and three biological replicates were performed. Total RNA from *V. dahliae* and *V. dahliae*-infected cotton plants was extracted with an E.Z.N.A.® Fungal RNA Kit. To analyse the expression of defense-related genes, one leaf of 4-week-old tobacco and cotton plants was treated with VdCP1 for every time point, and control plants were infiltrated with His-tag or BSA solution, 3 plants were treated for the assays and three biological replicates were performed. Total RNA was extracted from the indicated tissues at different time points using a plant RNA kit (TransGen Biotech). Amplification was performed in an ABI7500 Real-Time PCR system with TransStart Green qPCR SuperMix UDG (TransGen Biotech). All primers are listed in Table S1. The *V. dahliae*, tobacco and cotton actin genes, *vdActin* (XM_009660170), *NtEF1*α (XM_016658252) and *GhActin* (AY305737), respectively, were used to correct for sample-to-sample variation in the amount of RNA. The relative mRNA quantities were calculated from the threshold cycle using the ΔΔCt method (Schmittgen and Livak, 2008). Triplicate reactions were performed for all of the biological replicates to calculate the mean and deviation from the mean was calculated from the standard deviation.

### Assays for pathogen pathogenicity

Assays for the pathogenicity of the pathogens *P. syringae* pv. tabaci and *B. cinerea* were performed as previously described (Zhang et al., 2015). 4-week-old tobacco plant was infiltrated with VdCP1 on one side of the central vein of one leaf and cultured in the phytotron for 3 days, control plant was infiltrated with His-tag solution, 20 plants were treated for the assays and three biological replicates were carried out to calculate the average values. The treated leaves were then soaked in a *P. syringae* pv. tabaci suspension (OD_600_ = 0.05 in 10 mM MgCl_2_) with gentle swirling for 30 s. Three days after challenge with the bacteria, three punches were collected from each leaf and ground with a pestle in 1 ml of sterilized water. The sample suspensions were vortexed thoroughly and serially diluted to the appropriate dilution. The bacteria were subsequently spread on KB (Rif^50^) plates, and the plates were incubated for 48 h at 28°C. The number of colonies on each plate was then counted, the plates were photographed, and the degree of infection was calculated. For *B. cinerea* inoculation, the treated tobacco leaves were inoculated with 10 μl of the spore suspension (5 × 10^5^/ml in 60 mM KH_2_PO_4_, 10 mM glycine, 0.01% Tween 20, 0.1 M glucose) and cultured at 22°C under conditions of high humidity for 3 days. Lesions were observed at the indicated time points, and lesion sizes were calculated. Pathogenicity assays for the *vdcp1* WT, knockout and complementary strains were conducted using 2-week-old cotton seedlings. For each strain, conidial suspensions at 5 × 10^6^ /ml in sterile water were prepared. Cotton seedlings grown on soil in each pot and 15 pots were prepared for each strain, and this setup was replicated three times. 15 × 15 ml conidial suspension was pre-placed in 15 new trays respectively and each pot was fully immersed in the new tray until complete absorption. The inoculated seedlings were then grown for 2–3 weeks at 28°C, with a day/night period of 16/8 h. In addition, to test the ability of VdCP1 in inducing cotton disease resistance against *V. dahliae*, 2-week-old cotton seedlings were pre-infiltrated with VdCP1 on one cotyledon and cultured in the phytotron for 3 days, control plants were infiltrated with His-tag solution. The pre-treated seedlings were inoculated with WT conidial suspensions and grown as described above. The degree of wilt disease was divided into five grades as previously described: grade 0—healthy plant; grade 1—yellowing cotyledons; grade 2—wilting of the cotyledons and one true leaf; grade 3—wilting of all leaves and chlorosis; and grade 4—plant death (Liu et al., 2013). The control efficiency for *V. dahliae* was calculated using the following formulas: disease index value = [∑ (the number of seedling of every grade × relative grade)/ total seedlings × highest score (4)] × 100. Disease reduction (%) = [(disease index of control – disease index of treatment)/ disease index of control] × 100.

## Results

### Identification of secreted proteins

Many studies indicate that secretory proteomics offers an effective tool for exploring interesting fungus-plant interactions proteins (Medina et al., 2005; Kim et al., 2007; Paper et al., 2007; Shah et al., 2009; Espino et al., 2010). Although several secretory proteins from *V. dahliae* have been reported, few proteomic studies have been conducted on this fungus. Here, two integrated analyses of the *V. dahliae* early secretome were carried out individually. In total, 256 proteins, many of which were present due to cell lysis (Supplementary File 1), were identified in the supernatant. A number of putative pathogen-host interaction proteins in the secretome are highlighted and listed in Table 1, these proteins include cellulase, pectate lyase, chitinase, proteases, etc., as well as a potential effector (elicitor) protein that was analyzed from the Pathogen Host Interactions database (http://www.phi-base.org). It is meaningful to explore the roles of these interaction proteins in deciphering the pathogenic mechanism of *V. dahliae*.

**Table 1:** Putative pathogen-host interaction protein in the secretome.

**Gene ID/protein name/function**	**Signal P**
**Cellulase**	
VDAG_00511 | *Verticillium dahliae* VdLs.17 glucan 1,3-beta-glucosidase	Y
VDAG_02243 | *Verticillium dahliae* VdLs.17 1,3-beta-glucanosyltransferase gel1	Y
VDAG_02814 | *Verticillium dahliae* VdLs.17 glucan 1,3-beta-glucosidase	Y
VDAG_03896 | *Verticillium dahliae* VdLs.17 cellobiose dehydrogenase	Y
VDAG_03973 | *Verticillium dahliae* VdLs.17 beta-glucosidase	Y
VDAG_04035 | *Verticillium dahliae* VdLs.17 extracellular cell wall glucanase Crf1	Y
VDAG_04342 | *Verticillium dahliae* VdLs.17 mixed-linked glucanase	Y
VDAG_07168 | *Verticillium dahliae* VdLs.17 1,3-beta-glucanosyltransferase gel2	Y
VDAG_07192 | *Verticillium dahliae* VdLs.17 1,3-beta-glucanosyltransferase gel4	Y
VDAG_09510 | *Verticillium dahliae* VdLs.17 glucan 1,3-beta-glucosidase	Y
VDAG_09744 | *Verticillium dahliae* VdLs.17 glucan 1,3-beta-glucosidase	Y
VDAG_09750 | *Verticillium dahliae* VdLs.17 beta-glucosidase	Y
**Pectate lyase**	
VDAG_04977 | *Verticillium dahliae* VdLs.17 endopolygalacturonase	Y
VDAG_05545 | *Verticillium dahliae* VdLs.17 lipase	Y
VDAG_06155 | *Verticillium dahliae* VdLs.17 pectate lyase	Y
VDAG_07881 | *Verticillium dahliae* VdLs.17 pectinesterase	Y
VDAG_09063 | *Verticillium dahliae* VdLs.17 rhamnogalacturonase B	Y
**Chitinase**	
VDAG_04416 | *Verticillium dahliae* VdLs.17 chitinase	Y
VDAG_08741 | *Verticillium dahliae* VdLs.17 endochitinase	Y
**Proteases**	
VDAG_00500 | *Verticillium dahliae* VdLs.17 carboxypeptidase S1	Y
VDAG_00795 | *Verticillium dahliae* VdLs.17 zinc carboxypeptidase A 1	Y
VDAG_02304 | *Verticillium dahliae* VdLs.17 aminopeptidase Y	Y
VDAG_03555 | *Verticillium dahliae* VdLs.17 thymus-specific serine protease	Y
VDAG_04233 | *Verticillium dahliae* VdLs.17 aminopeptidase Y	Y
VDAG_04408 | *Verticillium dahliae* VdLs.17 dipeptidyl-peptidase	Y
VDAG_04979 | *Verticillium dahliae* VdLs.17 secreted aspartic proteinase	Y
VDAG_05455 | *Verticillium dahliae* VdLs.17 gamma-glutamyltranspeptidase	Y
VDAG_06386 | *Verticillium dahliae* VdLs.17 tripeptidyl-peptidase	Y
VDAG_07183 | *Verticillium dahliae* VdLs.17 carboxypeptidase A	Y
VDAG_07290 | *Verticillium dahliae* VdLs.17 carboxypeptidase B	Y
VDAG_07745 | *Verticillium dahliae* VdLs.17 bacterial leucyl aminopeptidase	Y
VDAG_10051 | *Verticillium dahliae* VdLs.17 tyrosinase	Y
**Effector (Elicitor)**	
VDAG_06199 | *Verticillium dahliae* VdLs.17 SnodProt1	Y

### Amino acid sequence analysis of VdCP1

Among these *V. dahliae*-cotton interaction proteins, one interesting SnodProt1 protein (VDAG_06199), designated VdCP1, was identified in the culture medium. VdCP1 contains 138 amino acids, has a predicted signal peptide at the cleavage site between amino acids 18 and 19 (SignalP 4.1 server), and is a typical CPP family homolog, exhibiting a low molecular weight, four conserved cysteine residues and high hydrophobicity. The mature VdCP1 protein contains 120 amino acid residues, has a predicted molecular mass of 12,607.1 Da. The amino acid sequence of VdCP1 indicated that it is highly homologous to Epl1, Sm1, BcSpl1 and CP (70.3, 68.1, 61.2, and 50.8%, respectively), which are among the best-characterized members of the CPP family (Figure S1). An amino acid sequence analysis of the post-translational modification sites using the NetOGlyc 4.0 and NetNGlyc 1.1 servers (http://www.cbs.dtu.dk) revealed the presence of potential O-glycosylation (residue 38, S) and N-glycosylation sites (residue 132, N), respectively, as indicated by the two arrows in Figure S1. In addition, hydropathicity plot (Kyte and Doolittle) analysis revealed that VdCP1 contains a high percentage of hydrophobic residues (Figure S1).

### Transcription profiles of *vdcp1* during the infection process

**Figure 1:**
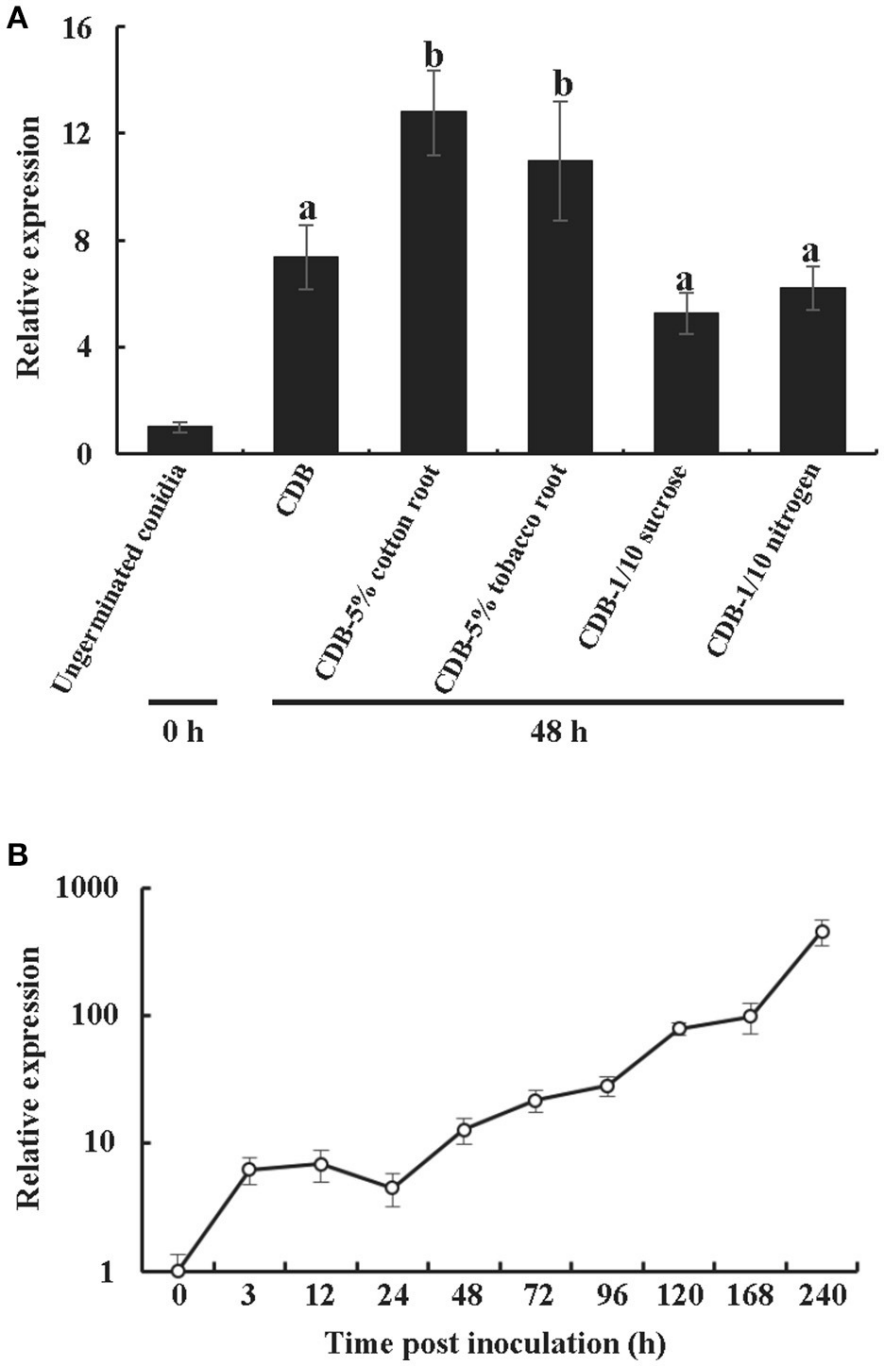
Expression profiles of *vdcp1* compared to ungerminated spores (0 h), as determined by qPCR. **(A)** The expression levels of *vdcp1* mRNA induced in different culture media with the indicated additions at 48 hpi. CDB with 5% cotton or tobacco roots means the basic CDB medium with the root fragments of two host plants added to 5% (w/v). CDB with 1/10 sucrose or nitrogen means a reduction in the content of sucrose and nitrogen, resulting in 1/10 sucrose or nitrogen in CDB. The letters above the bars indicate a statistically significant difference with ungerminated conidia at 0 h (*n* = 3, *p* < 0.05 by Tukey-Kramer's test). **(B)** The expression levels of *vdcp1* in *V. dahliae*-inoculated cotton stem base at the indicated time points are shown as the mRNA level relative to that in ungerminated spores. Triplicate biological replicates were used to determine the average values for quantification.

Like many previously characterized CPPs, VdCP1 was found in the culture medium. To obtain more details regarding the transcription of *vdcp1*, the *vdcp1* mRNA level in different culture mediums and at various stages of infection was measured via qPCR. The *vdcp1* mRNA levels increased with respect to ungerminated conidia in every medium studied at 48 h post-inoculation (hpi) (Figure 1A). In CDB culture medium supplemented with 5% cotton/tobacco roots, *vdcp1* mRNA levels showed relatively high upregulations, and *vdcp1* mRNA levels didn't show statistical differences between CDB and CDB with sucrose/nitrogen stress. During *V. dahliae* infection process, the amounts of *vdcp1* mRNA continuously increased and reached a maximum 456-fold increase over the levels observed at time zero (ungerminated conidia) (Figure 1B), thus we can conclude that *vdcp1* is expressed in the presence of all studied culture mediums and during all stages of the infection process, especially in planta during the late stages of infection.

### VdCP1 contributes to the pathogenicity of *V. dahliae*

**Figure 2:**
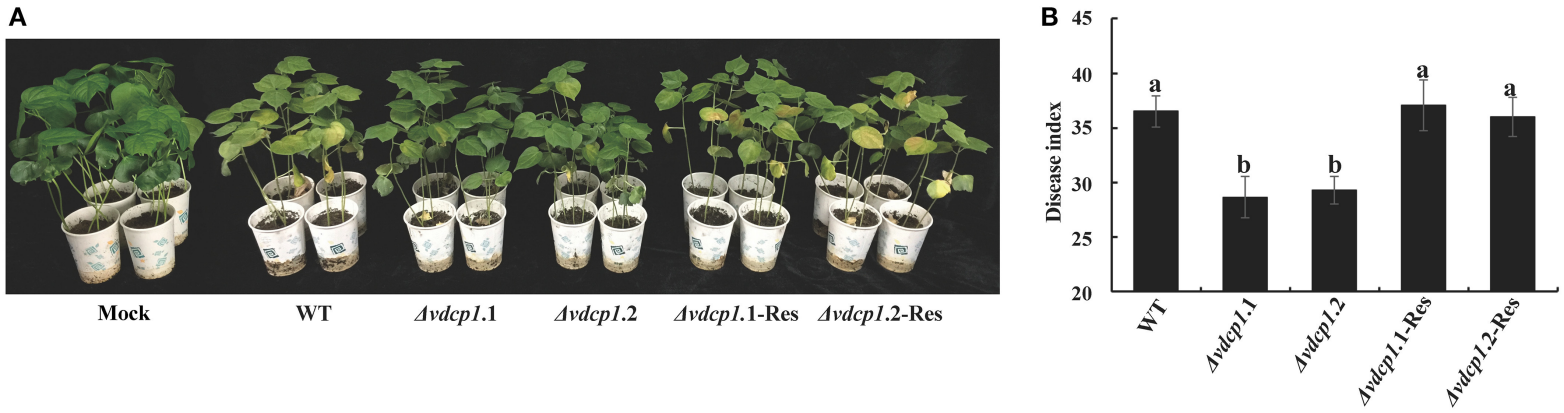
Assay for the pathogenicity of *V. dahliae* strains. **(A)** The example photographs show symptoms in cotton plants inoculated with the WT, Δ*vdcp1* and Δ*vdcp1-*Res strains. Mock was inoculated with sterile water. **(B)** The disease caused by *V. dahliae* strains as measured by the disease index. The letters above the bars indicate a statistically significant difference (*n* = 3, *p* < 0.05 by Tukey-Kramer's test).

To investigate the role of VdCP1 in the *V. dahliae* invasion process, the Δ*vdcp1* and Δ*vdcp1-*Res strains were constructed using the strategy shown in Figure S2. Two random transformants that exhibited hygromycin resistance and were unaffected by F2dU were selected. Genomic DNA and cDNA from both mutant strains were analyzed by PCR amplification, and all showed the expected bands (Figure S2). Moreover, qPCR using cDNA obtained from the transformants revealed no expression of *vdcp1* (data not shown), indicating that the *vdcp1* gene had been successfully knocked out in the selected transformants (Δ*vdcp1*.1 and Δ*vdcp1*.2). Complemented strains (Δ*vdcp1*.1-Res and Δ*vdcp1*.2-Res) were generated and characterized in a similar manner (Figure S2). The phenotypes of the Δ*vdcp1* and Δ*vdcp1*-Res strains with respect to their growth rate, sporulation capacity and mycelium/spore morphology were not discernibly different from those of the WT (Figure S3), whereas compared with the WT, both Δ*vdcp1* strains displayed a statistically significant decrease in their virulence to cotton, with nearly 20% infection, as calculated by the disease index, and the Δ*vdcp1*-Res strains showed restored virulence (Figures 2A,B).

### VdCP1 induces plant defense responses locally and systemically

Previous studies have shown that many CPPs are elicitors of plant defense responses. To test whether VdCP1 exhibits similar elicitation activity, *vdcp1* was inserted into the pPICZαA plasmid and expressed in *P. pastoris* KM71H. The purified VdCP1 showed a single band of 18 kDa upon SDS-PAGE and Western blotting and showed substantial matching with the VdCP1 sequence by mass spectrometry (MS) identification (Figure S4). Approximately 30 mg of VdCP1 was obtained from 100 ml of BMM culture supernatant.

**Figure 3:**
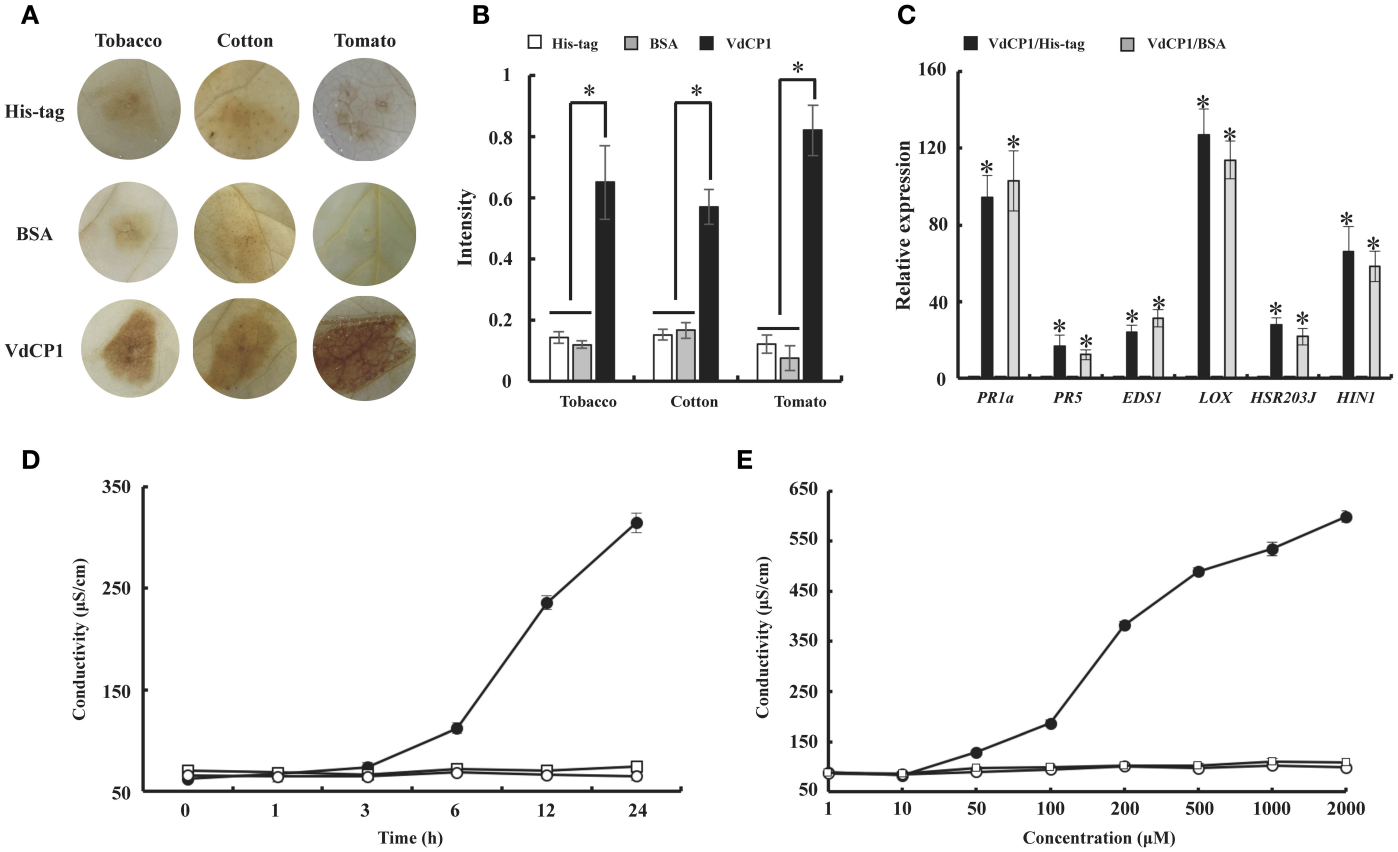
The induction of defense responses by VdCP1. **(A)** Induction of ROS in tobacco, tomato and cotton leaves after infiltration with 10 μM VdCP1, His-tag or BSA. The treated leaves were detached at 24 hpi and infiltrated with DAB solution. The presence of a brownish-red precipitate indicates the presence of hydrogen peroxide. **(B)** Quantification of DAB staining. Asterisks indicate a statistically significant difference (*p* < 0.05 by Student's *t*–test). **(C)** Induction of plant defense genes in tobacco leaves by treatment with 10 μM VdCP1, His-tag or BSA. Infiltrated leaves were cut at 24 hpi, and used to measure the transcript levels of *PR1a, PR5, LOX, EDS1, HSR203J*, and *HIN1*. Columns on the left of the black and gray columns are His-tag and BSA, respectively. Black columns correspond to VdCP1/His-tag (treatment/control), and gray columns correspond to VdCP1/BSA (treatment/control). Asterisks indicate a statistically significant difference (*p* < 0.05 by Student's *t*-test). **(D)** Tobacco leaves were infiltrated with 100 μM VdCP1 (closed circles), His-tag (open squares) or BSA (open circles). At 6 hpi, three leaf discs were punched from the infiltrated area and submerged in water, and the conductivity was measured at the indicated time points. **(E)** Tobacco leaves were infiltrated with the indicated concentrations of VdCP1 (closed circles), His-tag (open circles) or BSA (open squares). At 6 hpi, three leaf discs were punched from the infiltrated area and submerged in water, 6 h later, the conductivity was measured to produce a curve of concentration vs. conductivity. The results from three biological replicates were used to calculate the average values.

In general, an elicitor can be recognized by PRRs, and this recognition results in a series of characteristic symptoms such as the production of ROS, expression of defense genes and leakage of ion electrolytes (Chinchilla et al., 2009). Here, the production of hydrogen peroxide was detected using 3,3′-diaminobenzidine (DAB) staining. The result showed that VdCP1 (10 μM) triggered a marked increase in brown DAB precipitate in tobacco, cotton and tomato leaves (Figure 3A), and the intensity of signals with VdCP1 differed significantly from that with His-tag or BSA (Figure 3B). Moreover, the expression of 6 defense-related genes in treated tobacco leaves was elevated after infiltration with 10 μM VdCP1 solutions (His-tag or BSA were used as controls, respectively) (Figure 3C), these genes included (1) *PR-1a* (XM_009800164) and *PR5* (NM_001325216), which are pathogenesis-related genes (Spoel et al., 2009), (2) *LOX* (lipoxygenase, NM_001325784) (Veronesi et al., 1996) and *EDS1* (enhanced disease susceptibility 1, XM_016636310) (Rietz et al., 2011), which encode a key enzyme involved in the biosynthesis of jasmonic acid (JA) and a lipase that modulates salicylic acid (SA)-dependent disease resistance, respectively, as well as (3) *HSR203J* (XM_016619229) and *HIN1* (Y07563), which are two HR marker genes in tobacco (Pontier et al., 1994, 2001). Interestingly, two HR marker genes were induced (Figure 3C), however, no apparent necrosis appeared in the infiltration area at 24 hpi (data not shown). Additional assays using different concentrations of VdCP1 were conducted to further examine its necrosis-inducing activity. At concentrations of up to 200 μM, necrosis was observed in the infiltration area at 24 hpi (Figure S5). Cotton and tomato plants showed similar results (Figure S5), indicating that a high concentration of VdCP1 is required for HR-inducing activity. Additionally, the infiltration of tobacco leaves with VdCP1 (100 μM) also induced electrolyte leakage from the treated leaf tissue, as demonstrated by an increase in conductivity over time compared with that in control samples treated with His-tag or BSA at the same concentration (Figure 3D). When the assays were performed using different concentrations of VdCP1, the conductivity varied in a dose-dependent manner (Figure 3E). The minimum concentration of VdCP1 required to cause ion electrolyte leakage at 6 hpi was 50 μM.

**Figure 4:**
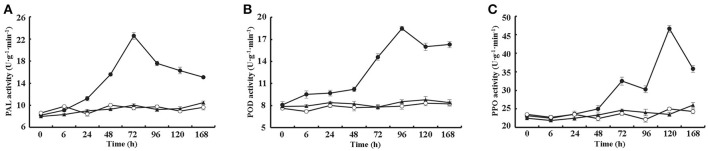
Kinetics of PAL, PPO, and POD activity after VdCP1 treatment of the cotton leaves. **(A–C)** PAL, POD, and PPO activities were measured at the indicated time points. Cotton leaves were treated with 10 μM VdCP1 (closed circles), His-tag (open circles) or BSA (closed triangles). Triplicate biological replicates were used to determine the average values.

Furthermore, the activities of three additional defense-related enzymes, PAL, POD and PPO, were assayed from 0 to 168 h after treating cotton leaves with 10 μM VdCP1, His-tag or BSA. The data showed that VdCP1 enhanced the activity of PAL, POD, and PPO, the activities of these enzymes peaked at 72, 96 and 120 h, respectively (Figures 4A–C).

**Figure 5:**
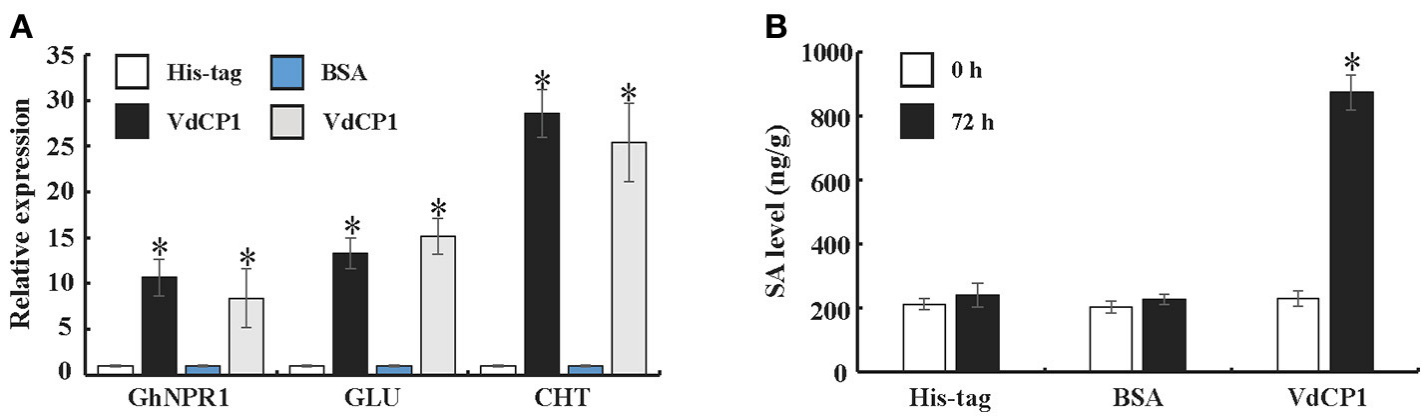
Systemic induction of defense genes and SA by infiltration with VdCP1. **(A)** Relative expression levels of *GhNPR1, GLU*, and *CHT* in untreated cotton roots at 72 h post inoculating cotyledons with 100 μM VdCP1, His-tag or BSA. **(B)** Systemic induction of SA levels in cotton roots by treating the cotyledons with 100 μM VdCP1, His-tag or BSA. Asterisks indicate a statistically significant difference (*p* < 0.05 by Student's *t*-test).

To further explore the ability of VdCP1 to induce systemic acquired resistance (SAR) in plants, a qPCR analysis was performed to analyse the expression of *GhNPR1* (a master gene for SAR, DQ325523) and two defense-related genes [PR genes, *GLU* (β-1,3-glucanase, CD486342) and *CHT* (chitinase, CD485880)] in cotton roots. The expression of *GhNPR1, GLU*, and *CHT* was significantly upregulated, respectively, at 72 h after inoculating cotyledons with VdCP1 (100 μM) compared with control plants treated with His-tag or BSA (Figure 5A). In the meantime, at 72 h after inoculating cotyledons with VdCP1 (100 μM), the SA levels in cotton roots increased prominently compared with those in control plants (Figure 5B).

### VdCP1 confers plants disease resistance

**Figure 6:**
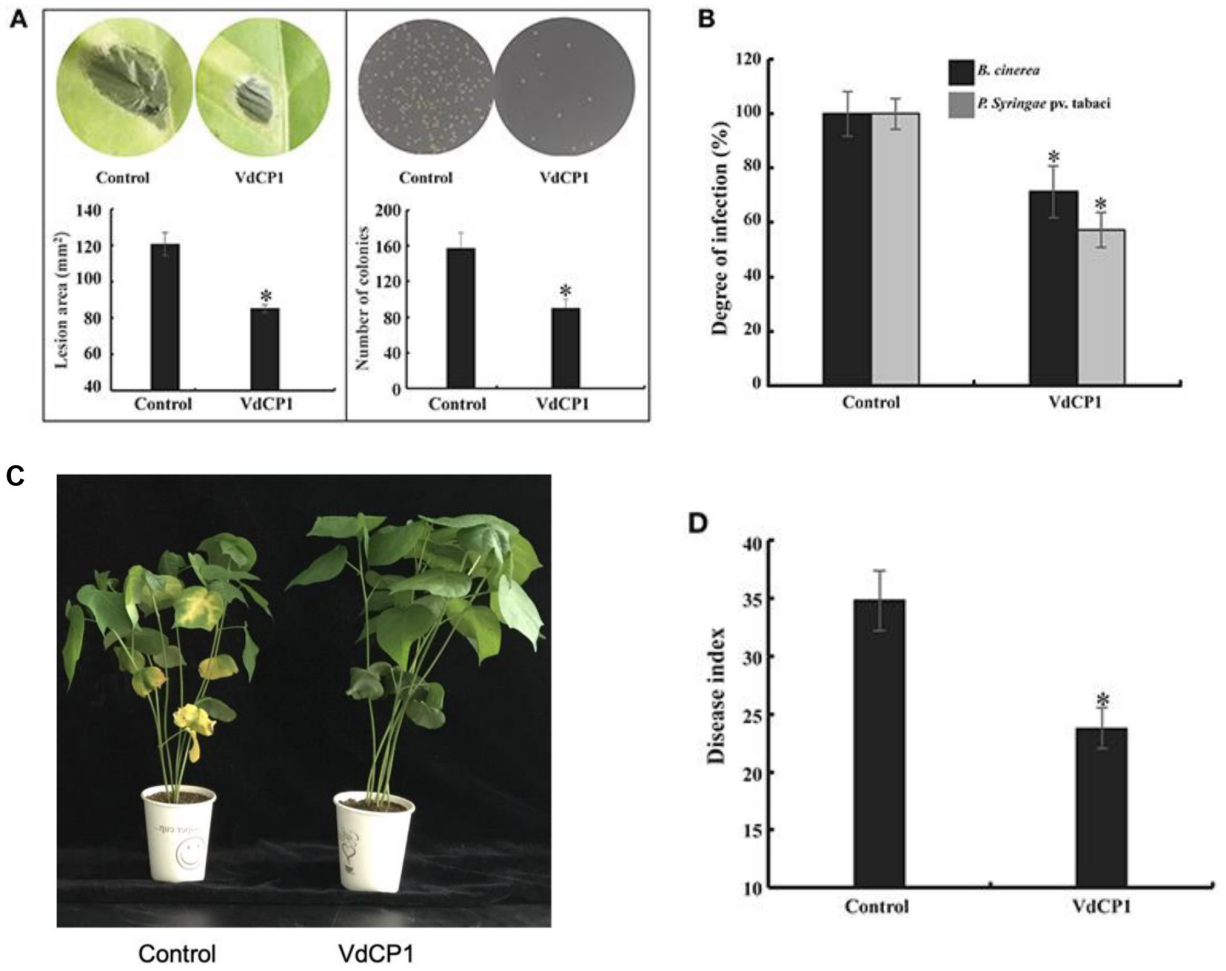
Induction of disease resistance against *P. syringae* pv. tabaci, *B. cinerea*, and *V. dahliae* in VdCP1-treated plants. **(A)** Tobacco leaves were treated with 100 μM VdCP1 or His-tag and inoculated with pathogens at 3 days after treatment, photographs were taken, then the *B. cinerea* lesion area in leaves and the number of the *P. syringae* colonies spread on plates were analyzed. **(B)** The induced tobacco plants showed resistance to the bacterial pathogen *P. syringae* pv. tabaci. The degree of infection was measured based on the number of bacteria obtained from the infected plants, and the degree of infection in the control was considered 100%. The induced tobacco plants also showed resistance to the fungal pathogen *B. cinerea*. The lesion area was calculated to determine the resistance of tobacco to *B. cinerea*. **(C)** Cotton seedlings induced with 100 μM VdCP1 or His-tag and infected with *V. dahliae*. The photographs show symptoms of cotton seedlings inoculated with *V. dahliae*. **(D)** Cotton seedlings treated with VdCP1 showed resistance to *V. dahliae*. The degree of infection is reflected by the disease index. Asterisks indicate a statistically significant difference (*p* < 0.05 by Student's *t*-test).

To explore whether VdCP1 could confer plants disease resistance, tobacco leaves infiltrated with 100 μM VdCP1 or His-tag were assayed for resistance against *B. cinerea* and *P. syringae* pv. tabaci. Remarkably, tobacco leaves pre-treated with VdCP1 showed disease resistance compared to the control plants. VdCP1-treated tobacco plants showed a significant reduction in the lesion area caused by *B. cinerea* on leaves (Figure 6A), the *B. cinerea* lesion area decreased by 29% (Figure 6B). The number of *P. syringae* pv. tabaci colonies of VdCP1-treated tobacco leaves on the plate was significantly lower than that of control leaves (Figure 6A), and the incidence of *P. syringae* pv. tabaci infection was as low as 43% that of the control (Figure 6B). In addition, cotton roots were inoculated with *V. dahliae* after 72 hpi of cotyledons with 100 μM VdCP1 or His-tag. After 2 weeks, wilt symptoms on the leaves of the VdCP1-induced and control cotton seedlings are shown in Figure 6C. The results showed a 32% reduction in the disease index compared with that of the control plants (Figure 6D).

### Effects of VdCP1 on polysaccharides and pathogens

**Figure 7:**
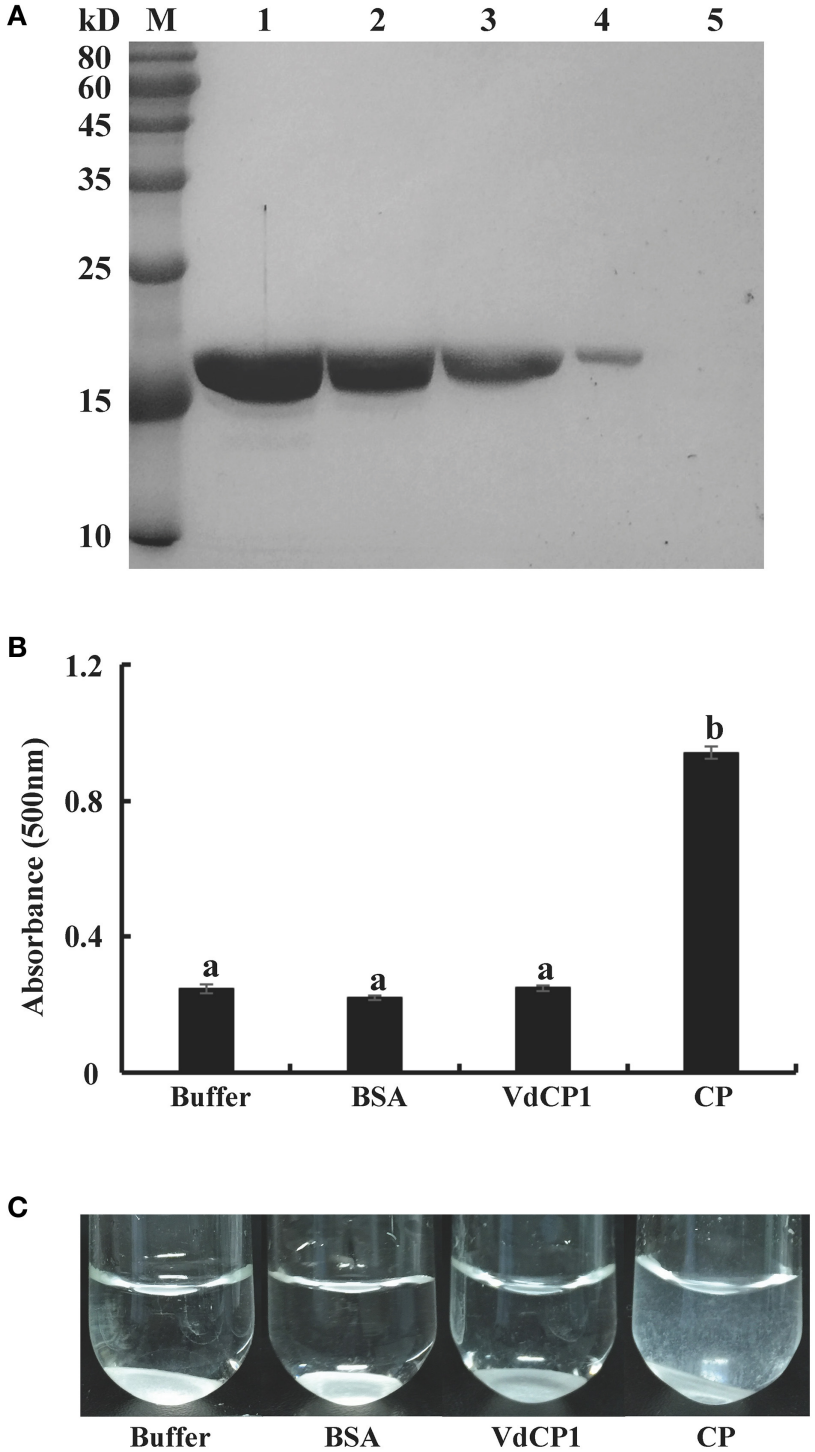
Assays of chitin-binding and filter paper-loosening activities of VdCP1. **(A)** VdCP1 was incubated with chitin from crab shells. All incubations were carried out in 50 mM sodium acetate buffer (1 ml, pH 6.5) at 38°C with shaking at 320 rpm. Unbound protein from the supernatant and bound protein from the pellet were analyzed via SDS-PAGE. M, protein molecular weight marker. 1, unbound protein with chitin, 2–4, bound protein with chitin (incubation for 12 h, 3 h and 5 min, respectively), 5, bound BSA with chitin (incubation for 5 min) **(B)** The absorbance of the suspension of paper fragments at 500 nm was measured via spectrophotometry after 2 days of incubation. Letters above the bars indicate a statistically significant difference (*n* = 3, *p* < 0.05 by Tukey-Kramer's test). **(C)** Photographs showing visible paper fragments released into suspension. Buffer and BSA at the same volume and/or concentration were included as negative controls, CP (10 μM) was used as a positive control.

The above results suggest that VdCP1 contributes to the pathogenicity of *V. dahliae* and elicits plant defense responses. Several CPPs with dual functions have been shown to bind to chitin and they show an expansin-like activity on cellulosic materials (Baccelli et al., 2013; Frischmann et al., 2013). To determine the chitin-binding activity of VdCP1, we performed experiments using chitin from crab shells. The results showed that VdCP1 could bind to chitin rapidly (5 min after incubation), while there was no binding activity between BSA and chitin (Figure 7A). The presence of protein CP on filter paper discs resulted in the appearance of paper fragments in the suspension (Baccelli et al., 2013), we then tested the expansin-like activity of VdCP1 on filter paper. No filter paper-loosening activity was detected for VdCP1 (Figure 7B), even at high concentrations of 200 μM (data not shown), however, CP (10 μM), which was used as the positive control, was able to loosen the filter paper significantly (Figure 7C).

The direct toxic activity of VdCP1 (100 μM) was tested separately with the fungal pathogen *B. cinerea* and the bacterial pathogen *P. syringae* pv. tabaci. No observable effects on growth inhibition were found (Figure S6). This result could rule out the possibility that VdCP1, which we used to induce plant disease resistance, has a direct negative effect on the growth of *B. cinerea* and *P. syringae* pv. tabaci.

### VdCP1 protected the *V. dahliae* cell wall

**Figure 8:**
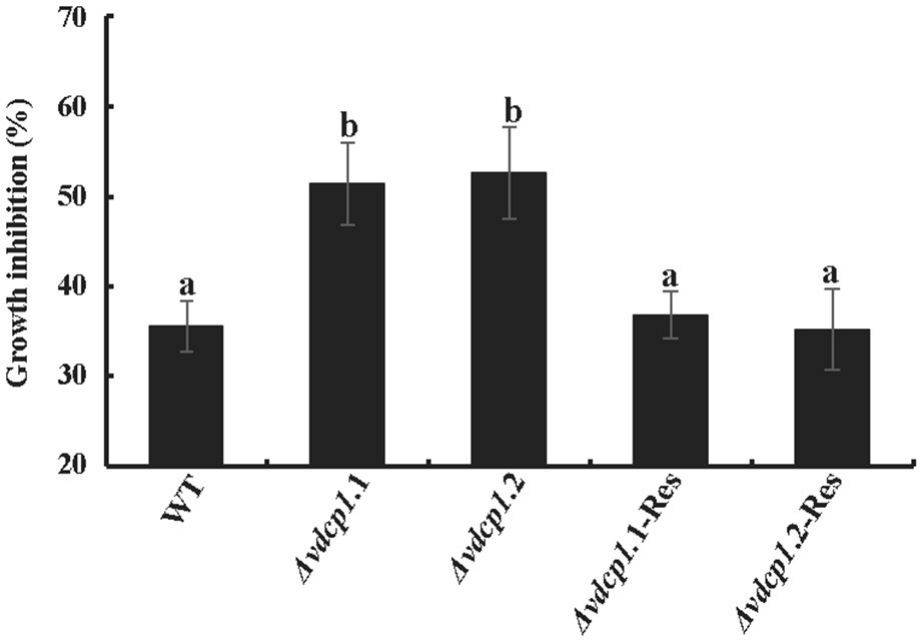
The sensitivity of *V. dahliae* WT, Δ*vdcp1* and Δ*vdcp1*-Res strains in the presence of 0.2 U of chitinase from *Streptomyces griseus*. Growth was determined spectrophotometrically by measuring the absorbance of the resazurin vital dye at 578 nm. Letters above the bars indicate statistically significant differences (*n* = 3, *p* < 0.05 by Tukey-Kramer's test).

Although no single phenotypic difference was observed in Δ*vdcp1* strains compared with WT, VdCP1 was shown to bind to chitin. To determine whether the absence of VdCP1 makes the cell wall of the Δ*vdcp1* mycelium more susceptible to enzymatic degradation by chitinase, the sensitivity of WT, Δ*vdcp1* and Δ*vdcp1*-Res strains to the presence of chitinase was studied. The result showed that chitinase could significantly affect the growth of all fungal strains, and the two mutant strains, Δ*vdcp1*.1 and Δ*vdcp1*.2, showed greater growth inhibition than the WT and Δ*vdcp1*-Res strains (Figure 8 and Figure S7), suggesting that VdCP1 can protect the *V. dahliae* cell wall from degradation by chitinase.

## Discussion

During the early stages of *V. dahliae* invasion, numerous proteins that facilitate the successful infection of host plants are secreted (Fradin and Thomma, 2006). Therefore, the *V. dahliae* secretome was carried out for identification of interesting proteins. In the present study, a SnodProt1-like protein (VDAG_06199), VdCP1, with 138 amino acids, a predicted molecular mass of 14,411.3 Da, was identified and characterized. This is the first report on a CPP in *V. dahliae*. We have shown here that VdCP1 is required for full virulence and that triggers the plant immune system.

The potential biological functions of CPPs are thought to involve fungal development and/or contribute to fungal virulence. The CPPs have been shown to participate in different growth stages (de O Barsottini et al., 2013; Frischmann et al., 2013). For example, among *epl1, epl2*, and *epl3* in *T. atroviride, epl1* is involved in hyphal growth and mycelial development, and *epl2* is expressed at the stage of spore maturation, whereas no *epl3* product has been detected to date (Frischmann et al., 2013). The expression of *B. cinerea bcspl1* and *M. grisea mgsm1* has been observed throughout all growth stages (Yang et al., 2009; Frías et al., 2011). In the present study, the expression level of *vdcp1* was continuously upregulated during invasion, even during spore germination (Figure 1B). The results suggest that *vdcp1* may play important roles throughout the growth process. Unfortunately, no single phenotypic difference between Δ*vdcp1* and WT strains was observed (Figure S3), thus, the characterization of the role of VdCP1 in fungal development awaits further research.

Several CPPs can bind to chitin and/or degrade cellulose (Baccelli et al., 2013; Frías et al., 2013; Frischmann et al., 2013). BcSpl1 can bind to the fungal cell wall, and *bcspl1* deletion significantly impairs the pathogenicity of *B. cinerea* (Frías et al., 2011; Frischmann et al., 2013). CP and Pop1 possess expansin-like activity, with a cellulosic material-loosening property (Baccelli et al., 2013). Furthermore, a previous study has found that Asp77 of CP is crucial for expansin-like and PAMP activities and is conserved throughout the members of CPPs (Luti et al., 2017), this Asp residue is also conserved in VdCP1. In our study, VdCP1 was shown to bind to chitin and could do so within 5 min of incubation (Figure 7A), but no effect on cellulosic material was detected (Figures 7B,C). A possible reason for these results is that the conserved Asp residue is an important factor for expansin-like and PAMP activities, but not the only one. BcSpl1 elicits defense responses in plants and has been proposed to be perceived as PAMPs by the plant immune system. The phytotoxic activity of BcSpl1 resides was found in a two-peptide motif on the protein surface, which didn't contain the conserved Asp residue, and two motifs including a disulfide bond of BcSpl1 are necessary and sufficient for its necrosis-inducing ability, and also suffice for its contribution to the virulence of the fungus, the truncated BcSpl1 proteins with the two-peptide motif could bind to tobacco protoplasts (Frías et al., 2013). It indicated that the two conserved Cys residues may be also significant for CPPs PAMP activity.

The chitin oligomers of the fungal cell wall can be recognized by LysM receptors, such as CERK1 in *Arabidopsis thaliana*, resulting in the activation of plant defense responses (Miya et al., 2007). Two LysM effectors, Slp1 from *M. oryzae* and Ecp6 from *C. fulvum*, contribute to virulence by combining with chitin oligomers and preventing the plant recognition of chitin oligomers (Miya et al., 2007; de Jonge et al., 2010; Mentlak et al., 2012). These properties support the hypothesis that VdCP1 may act by masking chitin oligomers, thereby attenuating host plant defense responses and contributing to *V. dahliae* infection. The reduction in the pathogenicity of the Δ*vdcp1* mutant suggested that VdCP1 possesses an effector function (Figure 2). In particular, the sensitivity of *V. dahliae* strains to a bacterial chitinase, which is similar to a PR protein produced in plant cells before and after infection (Van Loon et al., 2006), was tested, and the growth of Δ*vdcp1* mutants was apparently more inhibited that was that of the WT and Δ*vdcp1*-Res strains (Figure 8). VdCP1 protected the cell wall from chitinase degrading it into chitin oligomers, which can be perceived by LysM receptors. The ability to protect *V. dahliae* against bacterial chitinase may be an indication that the effector VdCP1 also protects against plants chitinases. We suggest that cell wall protection may be one of the reasons for the enhanced infection and the decreased virulence associated with *vdcp1* deletion.

Not all CPPs induce the necrosis in plants, as is the case for Epl1, Sm1 and MSP1 (Djonovic et al., 2006; Seidl et al., 2006; Jeong et al., 2007). Low concentrations of VdCP1 did not induce necrosis in treated plant leaves, however, the upregulation of *HSR203J* and *HIN1* were observed (Figure 3C). And necrosis resulted from the exposure of the leaves to high concentrations of VdCP1 (Figure S5). The possible explanations for these results are differences in the susceptibility of plants to VdCP1, as observed for the well-known elicitor ethylene-inducing xylanase (EIX), which induces weak necrosis in tobacco at a high concentration of 314 μM (Frías et al., 2011), or differences in activity among CPPs. Moreover, the inability of Epl1, Sm1 or MSP1 to induce necrosis may have resulted from insufficient concentration in the assay. In later studies, the transient expression of MSP1 and Sm1 in plants could induce the HR (Yang et al., 2009; Hong et al., 2017). On the other hand, *V. dahliae* is a hemibiotrophic fungus and differs from necrotrophs such as *B. cinerea*, which benefit from necrosis in achieving infection, therefore, the induction of necrosis might not be a direct phytotoxic effect of VdCP1 in *V. dahliae*. The CPPs from *M. grisea, Fusarium oxysporum, T. atroviride*, and *T. virens* all exhibited non-necrosis-inducing activity under low assay concentrations (Djonovic et al., 2006, Seidl et al., 2006, Jeong et al., 2007).

Although Epl1, Sm1 and MSP1 did not cause necrosis, they were able to induce the production of ROS and the expression of defense genes. ROS, produced as an early plant defense reaction against pathogens, are able to activate downstream defense responses (Zurbriggen et al., 2010). *PR* genes often are used as markers of plant response to pathogens and elicitors (Sels et al., 2008), and both *LOX* and *EDS1* are two key regulatory genes in the JA and SA pathways, respectively. Remarkably, VdCP1 induced the increase of ROS and upregulation of these defense-related genes (Figures 3A,C), resulting in resistance against *B. cinerea* and *P. syringae* (Figures 6A,B). Moreover, three important defense-related enzymes, PAL, POD, and PPO, were investigated in response to VdCP1. PAL and POD play important roles in the biosynthetic pathway of lignin, which can create a physical barrier to block the infection of pathogens. The significance of PPO in disease resistance comes from its ability to oxidize phenolic compounds to quinones, which are poisonous to pathogens. Accordingly, the increase in the activities of these enzymes can indicate plant disease resistance (De Ascensao and Dubery, 2000), which has also been observed in VdCP1-treated cotton plants (Figure 4). One of the hallmarks of SAR is the production of the plant hormone SA, which is necessary for SAR to occur. NPR1 is a key regulator in the signal transduction pathway that leads to SAR, which is able to induce PR genes in systemic tissues (Kinkema et al., 2000). GhNPR1 has been shown to play an important role in the response to signaling molecules in cotton plants (Zhang et al., 2008). In our study, SA levels, the transcription cofactor *GhNPR1* and two *PR* genes (*GLU* and *CHT*) were systemically induced in cotton root after inoculating cotyledon with VdCP1 (Figure 5), and exposure to VdCP1 conferred resistance to *V. dahliae* in cotton (Figures 6C,D). Our findings demonstrate that VdCP1 is involved in eliciting plant defense responses locally and systemically and that VdCP1 can improve the resistance of hosts to *B. cinerea, P. syringae*, and *V. dahliae*.

In summary, the low-molecular-weight protein VdCP1, which is a SnodProt1-like protein that belongs to the CPP family, was identified in the secretome of *V. dahliae*. VdCP1 is the first member of the CPP family to be described in *V. dahliae*. Our results seem to indicate that VdCP1 can protect the chitin component of the *V. dahliae* cell wall from enzymatic degradation, while it is shown to contribute to its virulence and to trigger the plant immune system.

## Author contributions

DQ and XY designed experiments. YZ carried out experiments and wrote the manuscript, YG analyzed experimental results. YL, YD, and JY assist the experiments.

### Conflict of interest statement

The authors declare that the research was conducted in the absence of any commercial or financial relationships that could be construed as a potential conflict of interest.

## Supplementary material

Figure S1 Sequence and hydropathicity plot analysis of VdCP1 homologs. **(A)** The GenBank accession numbers of VdCP1, Epl1, Sm1, BcSpl1, and CP are EGY15345.1, ABE73692.1, AAZ80388.1, CCD43517.1 and CAC84090.2, respectively. The conserved Cys residues are shown in gray outlined regions. Identical amino acid residues are indicated by asterisks, similar residues are indicated by dots, and glycosylation sites (O-glycosylation, residue 38, S and N-glycosylation sites, residue 132, N) are indicated by arrows. **(B)** Hydropathicity plot of VdCP1 (thick black line) and its homologs Epl1 (thin green line), Sm1 (thin red line), BcSpl1 (thin blue line) and CP (thin black line) were calculated using the method of Kyte and Doolittle.

Figure S2 Deletion and complementation of *vdcp1*. **(A,B)** Schematic map of the generation of the *vdcp1* knockout mutant and the *vdcp1* complementary strain, respectively. Dotted line means no transcription of *vdcp1* gene. **(C)** PCR analysis of the target transformant that was used for further experiments. For the detection of Δ*vdcp1* strains, extracted genomic DNA and synthesized cDNA were used as templates for PCR detection with the primers Check1 and Check2, respectively. To detect the Δ*vdcp1*-Res strains, extracted genomic DNA was used as a template for PCR detection with the primers Check3. Primers Check1 were designed from the 100-bp 5′ region and the 100-bp 3′ region of *vdcp1*, respectively. Primers Check2 were designed from the 5′ and 3′ region of *vdcp1* mRNA sequence. Primers Check3 are as same as primers Check1. M, marker. “Genome” indicates that the template used for PCR was genomic DNA from the target fungus, and “cDNA” indicates that the template was synthesized from mRNA obtained from the target fungus. Δ*vdcp1*.1 and Δ*vdcp1*.2 are the two *vdcp1* fungal deletion strains used in this study, Δ*vdcp1*.1-Re*s* and Δ*vdcp1*.2-Res are the two complementary strains corresponding to Δ*vdcp1*.1 and Δ*vdcp1*.2. WT is the background strain used to generate the *vdcp1* deletion.

Figure S3 The phenotypes of the Δ*vdcp1* and Δ*vdcp1*-Res strains with respect to their growth rate, sporulation capacity and mycelium/spore morphology compared with wild-type. **(A)** At 5 and 10 days after the inoculation of *V. dahliae* on PDA plates, the resultant colonies were photographed (5 days), and their diameters were measured. **(B)** Two weeks after the inoculation of *V. dahliae* on PDA plates, the spores were washed with water and filtered with Miracloth, and the spore concentration in the resulting suspension was measured using a haemocytometer. **(C)** The mycelium/spore morphology of the wild-type and mutant strains was observed via optical microscopy. Three biological replicates were performed in assays.

Figure S4 Purification and identification of expressed proteins. **(A,B)** Detection of purified VdCP1 and His-tag, respectively, via SDS-PAGE and Western blotting. M, protein molecular weight marker. Lanes 1 and 2 show the proteins expressed by KM71H after transformation with a control plasmid and with the recombinant plasmid, respectively. The proteins were stained with Coomassie Brilliant Blue R-250. Both Lane 2 show a single band for VdCP1 and His-tag, respectively. Lanes 3 and 4 contain the same samples as in Lanes 1 and 2, respectively, and were assayed by Western blotting with anti-His antibodies. Both Lane 4 show a single band, as well. **(C)** Mass spectrum analysis of recombinant VdCP1 protein. The amino acid residues shown in bold red indicate that the peptides digested by trypsin matched VdCP1.

Figure S5 Assay of the HR-inducing ability of VdCP1. Tobacco, cotton and tomato leaves were infiltrated with 200 μM of VdCP1, and photographs were also taken with front illumination at 24 h post infiltration to demonstrate the effect on the leaves. His-tag and BSA (at the same concentration of VdCP1) were controls.

Figure S6 The direct toxic activity of VdCP1 on pathogens. **(A)** At 5 days after the inoculation of *B. cinerea* on PDA plates containing 100 μM VdCP1 or His-tag, the growth rate of *B. cinerea* on PDA plates was calculated by measuring the diameter. **(B)** At 2 days after the inoculation of *P. syringae* pv. tabaci in KB Rif^100^ liquid medium containing 100 μM VdCP1 or His-tag, the growth of *P. syringae* pv. tabaci was calculated based on the OD_600_.

Figure S7 Color changes in PDB and the number of surviving spores. **(A)** Effect of the fungal spores on the color of PDB containing resazurin. PDB containing resazurin and chitinase without spores was blue, and PDB containing resazurin and chitinase with spores changed to pink, the color of the PDB with Δ*vdcp1* spores was deeper than that of the PDB with WT and Δ*vdcp1*-Res spores. PDB containing resazurin and spores without chitinase was light pink. **(B)** The PDB diluted appropriately in media was spread on PDA plates, and then the number of clones was calculated to show the spore survival. Letters above the bars indicate a statistically significant difference (*n* = 3, *p* < 0.05 by Tukey-Kramer's test).

## References

[B1] Baccelli I. Luti S. Bernardi R. Scala A. Pazzagli L. (2013). Cerato-platanin shows expansin-like activity on cellulosic materials. Appl. Microbiol. Biotechnol. 98, 175–184. 10.1007/s00253-013-4822-0 23512479

[B2] Boddi S. (2004). Cerato-platanin protein is located in the cell walls of ascospores, conidia and hyphae of *Ceratocystis fimbriata* f. sp. platani. FEMS Microbiol. Lett. 233, 341–346. 10.1111/j.1574-6968.2004.tb09501.x 15063505

[B3] Chang Y. H. Yan H. Z. Liou R. F. (2014). A novel elicitor protein from *Phytophthora parasitica* induces plant basal immunity and systemic acquired resistance. Mol. Plant Pathol. 16, 123–136. 10.1111/mpp.12166 24965864 PMC6638464

[B4] Chinchilla D. Shan L. He P. de Vries S. Kemmerling B. (2009). One for all: the receptor-associated kinase BAK1. Trends Plant Sci. 14, 535–541. 10.1016/j.tplants.2009.08.002 19748302 PMC4391746

[B5] De Ascensao A. R. Dubery I. A. (2000). Panama disease: cell wall reinforcement in banana roots in response to elicitors from *Fusarium oxysporum* f. sp. cubense race four. Phytopathology 90, 1173–1180. 10.1094/PHYTO.2000.90.10.1173 18944483

[B6] de Jonge R. van Esse H. P. Kombrink A. Shinya T. Desaki Y. Bours R.. (2010). Conserved fungal LysM effector Ecp6 prevents chitin-triggered immunity in plants. Science 329, 953–955. 10.1126/science.1190859 20724636

[B7] de O Barsottini M. R. de Oliveira J. F. Adamoski D. Teixeira P. J. P. L. do Prado P. F. V. Tiezzi H. O.. (2013). Functional diversification of cerato-platanins in *Moniliophthora perniciosaas* seen by differential expression and protein function specialization. Mol. Plant Microbe Interact. 26, 1281–1293. 10.1094/MPMI-05-13-0148-R 23902259

[B8] Djonovic S. Pozo M. J. Dangott L. J. Howell C. R. Kenerley C. M. (2006). Sm1, a proteinaceous elicitor secreted by the biocontrol fungus *Trichoderma virens* induces plant defense responses and systemic resistance. Mol. Plant Microbe Interact. 19, 838–853. 10.1094/MPMI-19-0838 16903350

[B9] Espino J. J. Gutiérrez-Sánchez G. Brito N. Shah P. Orlando R. González C. (2010). The *Botrytis cinerea* early secretome. Proteomics 10, 3020–3034. 10.1002/pmic.201000037 20564262 PMC3983782

[B10] Fradin E. F. Thomma B. P. H. J. (2006). Physiology and molecular aspects of Verticillium wilt diseases caused by *V. dahliae* and Valbo-atrum. Mol. Plant Pathol. 7, 71–86. 10.1111/j.1364-3703.2006.00323.x 20507429

[B11] Frías M. Brito N. González M. González C. (2013). The phytotoxic activity of the cerato-platanin BcSpl1 resides in a two-peptide motif on the protein surface. Mol. Plant Pathol. 15, 342–351. 10.1111/mpp.12097 24175916 PMC6638778

[B12] Frías M. González C. Brito N. (2011). BcSpl1, a cerato-platanin family protein, contributes to *Botrytis cinerea* virulence and elicits the hypersensitive response in the host. New Phytologist 192, 483–495. 10.1111/j.1469-8137.2011.03802.x 21707620

[B13] Frischmann A. Neudl S. Gaderer R. Bonazza K. Zach S. Gruber S.. (2013). Self-assembly at air/water interfaces and carbohydrate binding properties of the small secreted protein EPL1 from the fungus *Trichoderma atroviride*. J. Biol. Chem. 288, 4278–4287. 10.1074/jbc.M112.427633 23250741 PMC3567679

[B14] Gaderer R. Bonazza K. Seidl-Seiboth V. (2014). Cerato-platanins: a fungal protein family with intriguing properties and application potential. Appl. Microbiol. Biotechnol. 98, 4795–4803. 10.1007/s00253-014-5690-y 24687753 PMC4024134

[B15] González-Fernández R. Aloria K. Valero-Galván J. Redondo I. Arizmendi J. M. Jorrín-Novo J. V. (2014). Proteomic analysis of mycelium and secretome of different *Botrytis cinerea* wild-type strains. J. Proteomics 97, 195–221. 10.1016/j.jprot.2013.06.022 23811051

[B16] Hano C. Addi M. Fliniaux O. Bensaddek L. Duverger E. Mesnard F.. (2008). Molecular characterization of cell death induced by a compatible interaction between *Fusarium oxysporum* f. sp. linii and flax (*Linum usitatissimum)* cells. Plant Physiol. Biochem. 46, 590–600. 10.1016/j.plaphy.2008.02.004 18396055

[B17] Hong Y. Yang Y. Zhang H. Huang L. Li D. Song F. (2017). Overexpression of *MoSM1*, encoding for an immunity-inducing protein from *Magnaporthe oryzae*, in rice confers broad-spectrum resistance against fungal and bacterial diseases. Sci. Rep. 7:41037. 10.1038/srep41037 28106116 PMC5247740

[B18] Jeong J. S. Mitchell T. K. Dean R. A. (2007). The *Magnaporthe grisea* snodprot1 homolog, MSP1, is required for virulence. FEMS Microbiol. Lett. 273, 157–165. 10.1111/j.1574-6968.2007.00796.x 17590228

[B19] Kim Y. Nandakumar M. P. Marten M. R. (2007). Proteomics of filamentous fungi. Trends Biotechnol. 25, 395–400. 10.1016/j.tibtech.2007.07.008 17681627

[B20] Kinkema M. Fan W. Dong X. (2000). Nuclear localization of NPR1 is required for activation of PR gene expression. Plant Cell Online 12, 2339–2350. 10.1105/tpc.12.12.2339 11148282 PMC102222

[B21] Klosterman S. J. Atallah Z. K. Vallad G. E. Subbarao K. V. (2009). Diversity, pathogenicity, and management of *Verticillium* Species. Annu. Rev. Phytopathol. 47, 39–62. 10.1146/annurev-phyto-080508-081748 19385730

[B22] Liu S. Y. Chen J. Y. Wang J. L. Li L. Xiao H. L. Adam S. M.. (2013). Molecular characterization and functional analysis of a specific secreted protein from highly virulent defoliating *Verticillium dahliae*. Gene 529, 307–316. 10.1016/j.gene.2013.06.089 23891822

[B23] Luti S. Martellini F. Bemporad F. Mazzoli L. Paoli P. Pazzagli L. (2017). A single amino acid mutation affects elicitor and expansins-like activities of cerato-platanin, a non-catalytic fungal protein. PLoS ONE 12:e0178337. 10.1371/journal.pone.0178337 28542638 PMC5444802

[B24] Lv S. Wang Z. Yang X. Guo L. Qiu D. Zeng H. (2016). Transcriptional profiling of rice treated with mohrip1 reveal the function of protein elicitor in enhancement of disease resistance and plant growth. Front. Plant Sci. 7:1818. 10.3389/fpls.2016.01818 27990152 PMC5131010

[B25] Medina M. L. Haynes P. A. Breci L. Francisco W. A. (2005). Analysis of secreted proteins from *Aspergillus flavus*. Proteomics 5, 3153–3161. 10.1002/pmic.200401136 16035112

[B26] Mengiste T. (2012). Plant immunity to necrotrophs. Annu. Rev. Phytopathol. 50, 267–294. 10.1146/annurev-phyto-081211-172955 22726121

[B27] Mentlak T. A. Kombrink A. Shinya T. Ryder L. S. Otomo I. Saitoh H.. (2012). Effector-mediated suppression of chitin-triggered immunity by *Magnaporthe oryzae* is necessary for rice blast disease. Plant Cell 24, 322–335. 10.1105/tpc.111.092957 22267486 PMC3289562

[B28] Miya A. Albert P. Shinya T. Desaki Y. Ichimura K. Shirasu K.. (2007). CERK1, a LysM receptor kinase, is essential for chitin elicitor signaling in *Arabidopsis*. Proc. Natl. Acad. Sci. U.S.A. 104, 19613–19618. 10.1073/pnas.0705147104 18042724 PMC2148337

[B29] Paper J. M. Scott-Craig J. S. Adhikari N. D. Cuomo C. A. Walton J. D. (2007). Comparative proteomics of extracellular proteins *in vitro* and in planta from the pathogenic fungus *Fusarium graminearum*. Proteomics 7, 3171–3183. 10.1002/pmic.200700184 17676664

[B30] Pazzagli L. Cappugi G. Manao G. Camici G. Santini A. Scala A. (1999). Purification, characterization, and amino acid sequence of cerato-platanin, a new phytotoxic protein from *Ceratocystis fimbriata* f. sp. platani. J. Biol. Chem. 274, 24959–24964. 10.1074/jbc.274.35.24959 10455173

[B31] Pontier D. Balagu,é C. Bezombes-Marion I. Tronchet M. Deslandes L. Roby D. (2001). Identification of a novel pathogen-responsive element in the promoter of the tobacco gene HSR203J, a molecular marker of the hypersensitive response. Plant J. 26, 495–507. 10.1046/j.1365-313x.2001.01049.x 11439136

[B32] Pontier D. Godiard L. Marco Y. Roby D. (1994). hsr203J, a tobacco gene whose activation is rapid, highly localized and specific for incompatible plant/pathogen interactions. Plant J. 5, 507–521. 10.1046/j.1365-313X.1994.5040507.x 8012404

[B33] Quarantin A. Glasenapp A. Schäfer W. Favaron F. Sella L. (2016). Involvement of the *Fusarium graminearum* cerato-platanin proteins in fungal growth and plant infection. Plant Physiol. Biochem. 109, 220–229. 10.1016/j.plaphy.2016.10.001 27744264

[B34] Rietz S. Stamm A. Malonek S. Wagner S. Becker D. Medina-Escobar N.. (2011). Different roles of Enhanced Disease Susceptibility1 (EDS1) bound to and dissociated from Phytoalexin Deficient4 (PAD4) in *Arabidopsis* immunity. New Phytol. 191, 107–119. 10.1111/j.1469-8137.2011.03675.x 21434927

[B35] Scala A. Pazzagli L. Comparini C. Santini A. Tegli S. Cappugi G. (2004). Cerato-platanin, an early-produced protein by *Ceratocystis fimbriata* f. sp. platani, elicits phytoalexin synthesis in host and non-host plants. J. Plant Pathol. 86, 23–29. 10.4454/jpp.v86i1.934

[B36] Schmittgen T. D. Livak K. J. (2008). Analyzing real-time PCR data by the comparative CT method. Nat. Protoc. 3, 1101–1108. 10.1038/nprot.2008.73 18546601

[B37] Seidl V. Marchetti M. Schandl R. Allmaier G. Kubicek C. P. (2006). Epl1, the major secreted protein of *Hypocrea atroviridis* on glucose, is a member of a strongly conserved protein family comprising plant defense response elicitors. FEBS J. 273, 4346–4359. 10.1111/j.1742-4658.2006.05435.x 16939625

[B38] Sella L. Gazzetti K. Castiglioni C. Schäfer W. Favaron F. (2014). *Fusarium graminearum* possesses virulence factors common to fusarium head blight of wheat and seedling rot of soybean but differing in their impact on disease severity. Phytopathology 104, 1201–1207. 10.1094/PHYTO-12-13-0355-R 24779355

[B39] Sels J. Mathys J. De Coninck B. M. A. Cammue B. P. A. De Bolle M. F. C. (2008). Plant pathogenesis-related (PR) proteins: a focus on PR peptides. Plant Physiol. Biochem. 46, 941–950. 10.1016/j.plaphy.2008.06.011 18674922

[B40] Shah P. Atwood J. A. Orlando R. Mubarek E. l. H. Podila G. K. Davis M. R. (2009). Comparative proteomic analysis of *Botrytis cinerea* secretome. J. Proteome Res. 8, 1123–1130. 10.1021/pr8003002 19140674

[B41] Shi F. M. Li Y. Z. (2008). *Verticillium dahliae* toxins-induced nitric oxide production in *Arabidopsis* is major dependent on nitrate reductase. BMB Rep. 41, 79–85. 10.5483/BMBRep.2008.41.1.079 18304455

[B42] Skinner W. Keon J. Hargreaves J. (2001). Gene information for fungal plant pathogens from expressed sequences. Curr. Opin. Microbiol. 4, 381–386. 10.1016/S1369-5274(00)00221-6 11495798

[B43] Spoel S. H. Mou Z. Tada Y. Spivey N. W. Genschik P. Dong X. (2009). Proteasome-mediated turnover of the transcription coactivator NPR1 plays dual roles in regulating plant immunity. Cell 137, 860–872. 10.1016/j.cell.2009.03.038 19490895 PMC2704463

[B44] Van Loon L. C. Rep M. Pieterse C. M. J. (2006). Significance of inducible defense-related proteins in infected plants. Annu. Rev. Phytopathol. 44, 135–162. 10.1146/annurev.phyto.44.070505.143425 16602946

[B45] Veronesi C. Rickauer M. Fournier J. Pouenat M. L. Esquerre-Tugaye M. T. (1996). Lipoxygenase gene expression in the tobacco-*Phytophthora parasitica* nicotianae interaction. Plant Physiol. 112, 997–1004. 10.1104/pp.112.3.997 8938408 PMC158026

[B46] Wang B. Yang X. Zeng H. Liu H. Zhou T. Tan B.. (2011). The purification and characterization of a novel hypersensitive-like response-inducing elicitor from *Verticillium dahliae* that induces resistance responses in tobacco. Appl. Microbiol. Biotechnol. 93, 191–201. 10.1007/s00253-011-3405-1 21691787

[B47] Wang X. Li X. Deng X. Han H. Shi W. Li Y. (2007). A protein extraction method compatible with proteomic analysis for the euhalophyte *Salicornia europaea*. Electrophoresis 28, 3976–3987. 10.1002/elps.200600805 17960840

[B48] Yang Y. Zhang H. Li G. Li W. Wang X. Song F. (2009). Ectopic expression of MgSM1, a Cerato-platanin family protein from *Magnaporthe grisea*, confers broad-spectrum disease resistance in *Arabidopsis*. Plant Biotechnol. J. 7, 763–777. 10.1111/j.1467-7652.2009.00442.x 19754836

[B49] Zhang Y. Liang Y. Dong Y. Gao Y. Yang X. Yuan J.. (2017a). The *Magnaporthe oryzae* Alt A 1-like protein MoHrip1 binds to the plant plasma membrane. Biochem. Biophys. Res. Commun. 492, 55–60. 10.1016/j.bbrc.2017.08.039 28807829

[B50] Zhang Y. Liang Y. Qiu D. Yuan J. Yang X. (2017b). Comparison of cerato-platanin family protein BcSpl1 produced in *Pichia pastoris* and *Escherichia coli*. Protein Expr. Purif. 136, 20–26. 10.1016/j.pep.2017.06.004 28606662

[B51] Zhang Y. Wang X. Cheng C. Gao Q. Liu J. Guo X. (2008). Molecular cloning and characterization of GhNPR1, a gene implicated in pathogen responses from cotton (*Gossypium hirsutum* L.). Biosci. Rep. 28, 7–9. 10.1042/BSR20070028 18215146

[B52] Zhang Y. Yang X. Liu Q. Qiu D. Zhang Y. Zeng H.. (2010). Purification of novel protein elicitor from *Botrytis cinerea* that induces disease resistance and drought tolerance in plants. Microbiol. Res. 165, 142–151. 10.1016/j.micres.2009.03.004 19616421

[B53] Zhang Y. Zhang Y. Qiu D. Zeng H. Guo L. Yang X. (2015). BcGs1, a glycoprotein from *Botrytis cinerea*, elicits defence response and improves disease resistance in host plants. Biochem. Biophys. Res. Commun. 457, 627–634. 10.1016/j.bbrc.2015.01.038 25613865

[B54] Zurbriggen M. D. Carrillo N. Hajirezaei M. R. (2010). ROS signaling in the hypersensitive response: when, where and what for? Plant Signal. Behav. 5, 393–396. 10.4161/psb.5.4.10793 20383072 PMC2958590

