# Correction: The *Verticillium dahliae* SnodProt1-like protein VdCP1 contributes to virulence and triggers the plant immune system

**DOI:** 10.3389/fpls.2025.1682576

**Published:** 2025-09-09

**Authors:** Yi Zhang, Yuhan Gao, Yingbo Liang, Yijie Dong, Xiufen Yang, Jingjing Yuan, Dewen Qiu

**Affiliations:** Institute of Plant Protection (CAAS), Haidian, China

**Keywords:** *Verticillium dahlia*, cerato-platanin, SnodProt1, virulence, elicitor, plant immunity

In the original article, there was a mistake in **Figure 6C** as published. It caught the authors’ attention that the picture of disease symptom phenotype in the original **Figure 6C** was mistakenly introduced during the figure preparation. The error occurred because the duplicated photos taken during early-stage pathogen infection experiments were not immediately and correctly marked and were later mistaken for another experiment when preparing the figures. The corrected **Figure 6** appears below.

**Figure 6:**
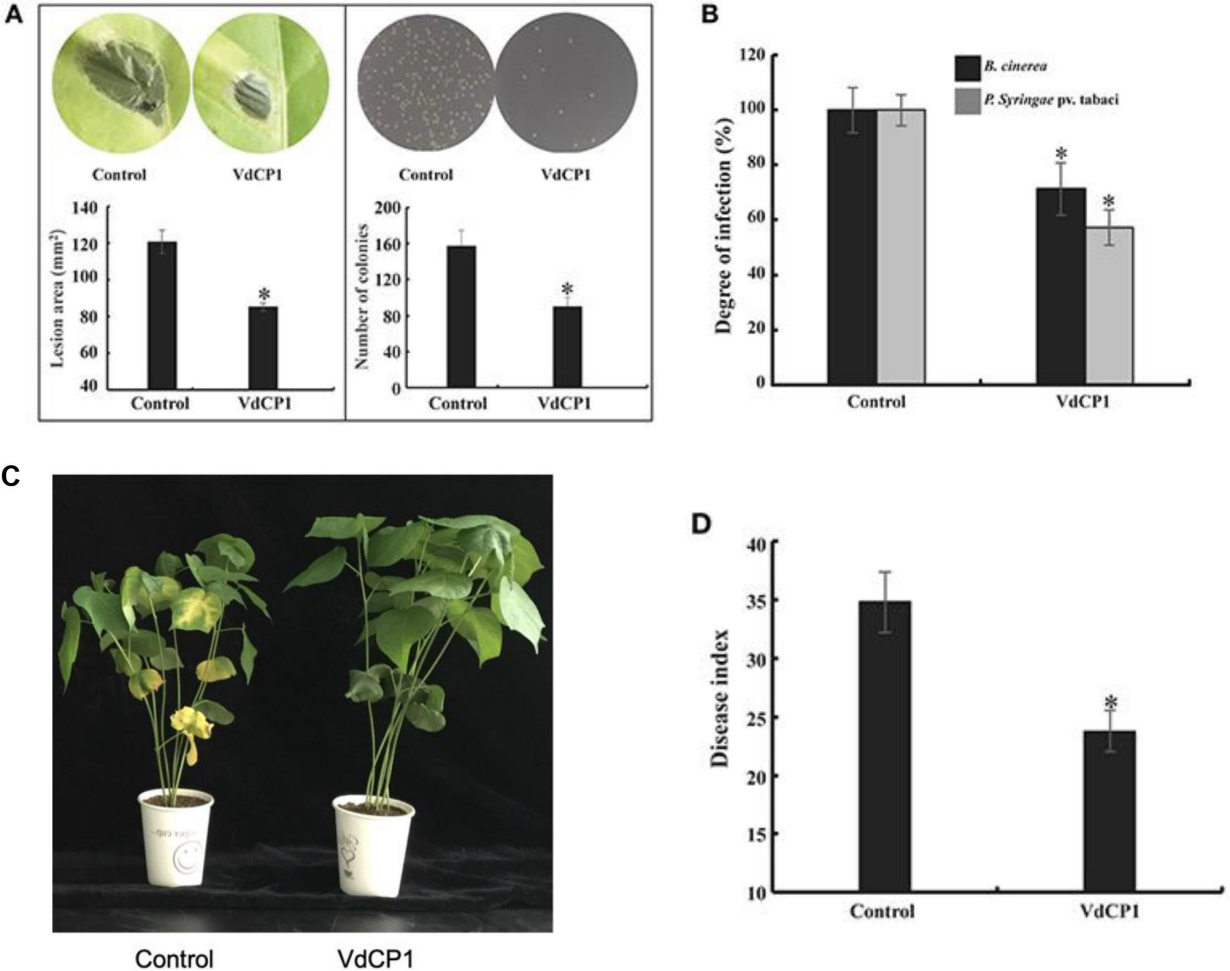
Induction of disease resistance against *P. syringae* pv. tabaci, *B. cinerea*, and *V. dahliae* in VdCP1-treated plants. **(A)** Tobacco leaves were treated with 100 μM VdCP1 or His-tag and inoculated with pathogens at 3 days after treatment, photographs were taken, then the *B. cinerea* lesion area in leaves and the number of the *P. syringae* colonies spread on plates were analyzed. **(B)** The induced tobacco plants showed resistance to the bacterial pathogen *P. syringae* pv. tabaci. The degree of infection was measured based on the number of bacteria obtained from the infected plants, and the degree of infection in the control was considered 100%. The induced tobacco plants also showed resistance to the fungal pathogen *B. cinerea*. The lesion area was calculated to determine the resistance of tobacco to *B. cinerea*. **(C)** Cotton seedlings induced with 100 µM VdCP1 or His-tag (control) and infected with *V. dahliae*. The photographs show symptoms of cotton seedlings inoculated with *V. dahliae*. **(D)** Cotton seedlings treated with VdCP1 showed resistance to *V. dahliae*. The degree of infection is reflected by the disease index. Asterisks indicate a statistically significant difference (p < 0.05 by Student's t-test).

The original article has been updated.

