# Supplementary figures and images for: The Verticillium dahliae SnodProt1-Like Protein VdCP1 Contributes to Virulence and Triggers the Plant Immune System
Source: Front Plant Sci. 2017 Oct 31;8:1880. doi: 10.3389/fpls.2017.01880 (PMC5671667; doi:10.3389/fpls.2017.01880)

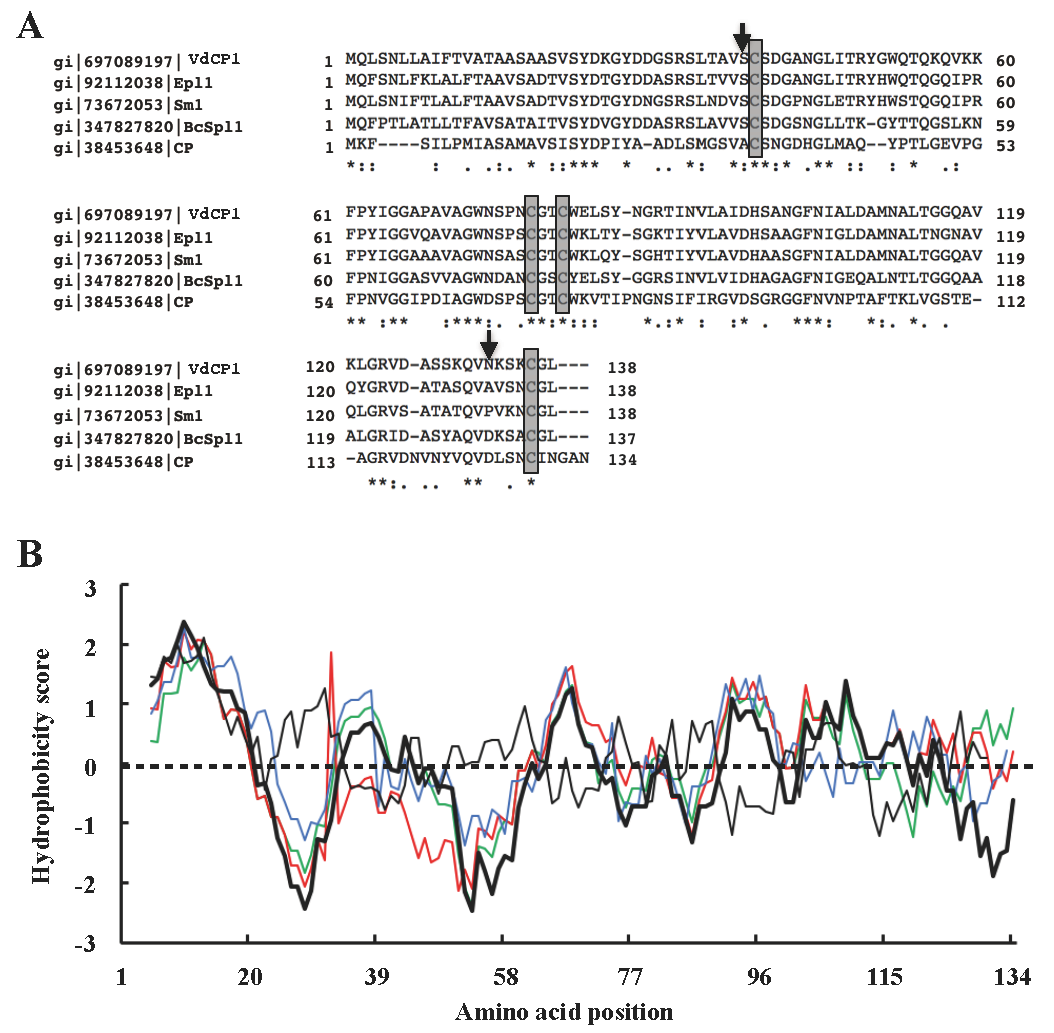

Supplement: Figure S1 — Sequence and hydropathicity plot analysis of VdCP1 homologs. (A) The GenBank accession numbers of VdCP1, Epl1, Sm1, BcSpl1, and CP are EGY15345.1, ABE73692.1, AAZ80388.1, CCD43517.1 and CAC84090.2, respectively. The conserved Cys residues are shown in gray outlined regions. Identical amino acid residues are indicated by asterisks, similar residues are indicated by dots, and glycosylation sites (O-glycosylation, residue 38, S and N-glycosylation sites, residue 132, N) are indicated by arrows. (B) Hydropathicity plot of VdCP1 (thick black line) and its homologs Epl1 (thin green line), Sm1 (thin red line), BcSpl1 (thin blue line) and CP (thin black line) were calculated using the method of Kyte and Doolittle. [file Image1.TIFF]

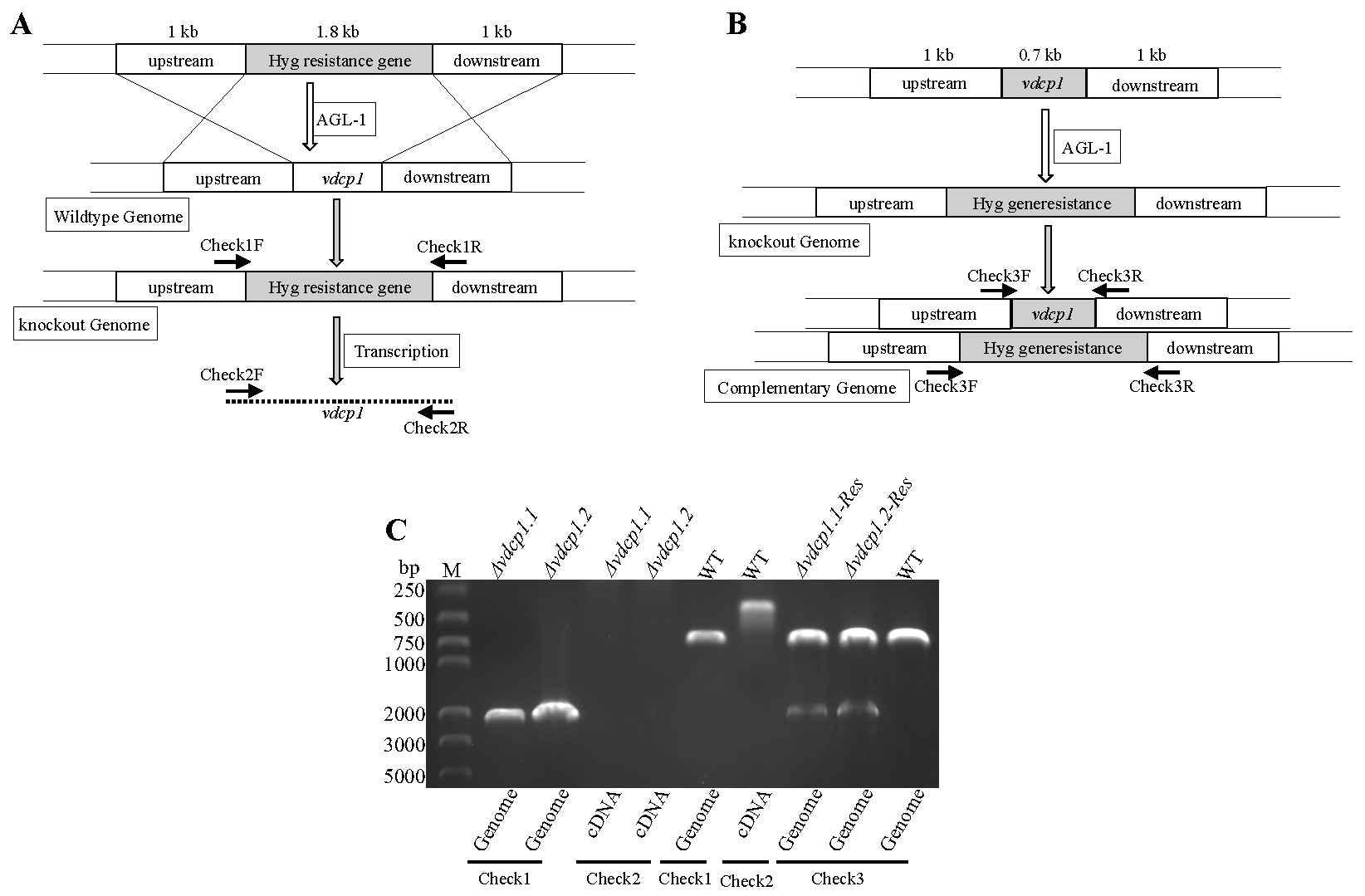

Supplement: Figure S2 — Deletion and complementation of vdcp1. (A,B) Schematic map of the generation of the vdcp1 knockout mutant and the vdcp1 complementary strain, respectively. Dotted line means no transcription of vdcp1 gene. (C) PCR analysis of the target transformant that was used for further experiments. For the detection of Δvdcp1 strains, extracted genomic DNA and synthesized cDNA were used as templates for PCR detection with the primers Check1 and Check2, respectively. To detect the Δvdcp1-Res strains, extracted genomic DNA was used as a template for PCR detection with the primers Check3. Primers Check1 were designed from the 100-bp 5′ region and the 100-bp 3′ region of vdcp1, respectively. Primers Check2 were designed from the 5′ and 3′ region of vdcp1 mRNA sequence. Primers Check3 are as same as primers Check1. M, marker. “Genome” indicates that the template used for PCR was genomic DNA from the target fungus, and “cDNA” indicates that the template was synthesized from mRNA obtained from the target fungus. Δvdcp1.1 and Δvdcp1.2 are the two vdcp1 fungal deletion strains used in this study, Δvdcp1.1-Res and Δvdcp1.2-Res are the two complementary strains corresponding to Δvdcp1.1 and Δvdcp1.2. WT is the background strain used to generate the vdcp1 deletion. [file Image2.TIFF]

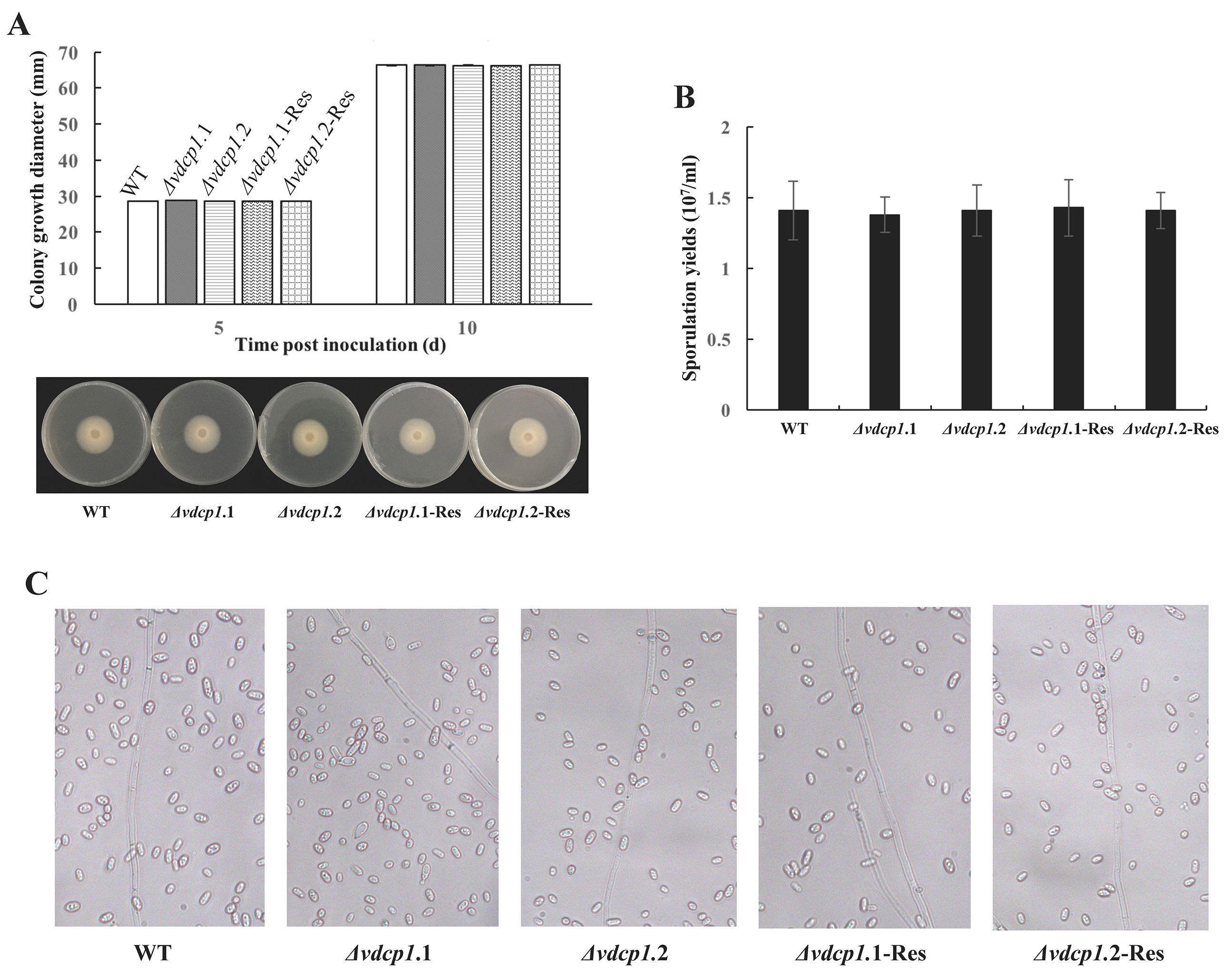

Supplement: Figure S3 — The phenotypes of the Δvdcp1 and Δvdcp1-Res strains with respect to their growth rate, sporulation capacity and mycelium/spore morphology compared with wild-type. (A) At 5 and 10 days after the inoculation of V. dahliae on PDA plates, the resultant colonies were photographed (5 days), and their diameters were measured. (B) Two weeks after the inoculation of V. dahliae on PDA plates, the spores were washed with water and filtered with Miracloth, and the spore concentration in the resulting suspension was measured using a haemocytometer. (C) The mycelium/spore morphology of the wild-type and mutant strains was observed via optical microscopy. Three biological replicates were performed in assays. [file Image3.JPEG]

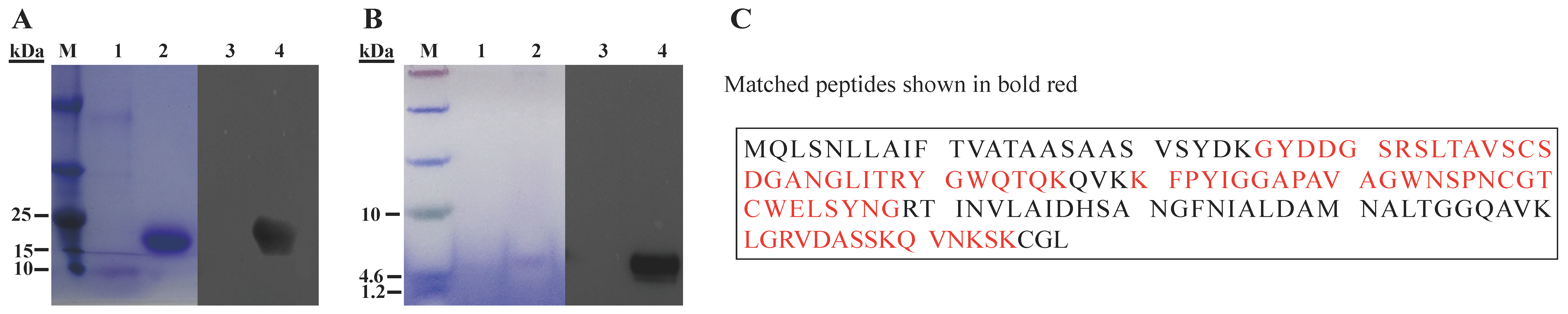

Supplement: Figure S4 — Purification and identification of expressed proteins. (A,B) Detection of purified VdCP1 and His-tag, respectively, via SDS-PAGE and Western blotting. M, protein molecular weight marker. Lanes 1 and 2 show the proteins expressed by KM71H after transformation with a control plasmid and with the recombinant plasmid, respectively. The proteins were stained with Coomassie Brilliant Blue R-250. Both Lane 2 show a single band for VdCP1 and His-tag, respectively. Lanes 3 and 4 contain the same samples as in Lanes 1 and 2, respectively, and were assayed by Western blotting with anti-His antibodies. Both Lane 4 show a single band, as well. (C) Mass spectrum analysis of recombinant VdCP1 protein. The amino acid residues shown in bold red indicate that the peptides digested by trypsin matched VdCP1. [file Image4.TIFF]

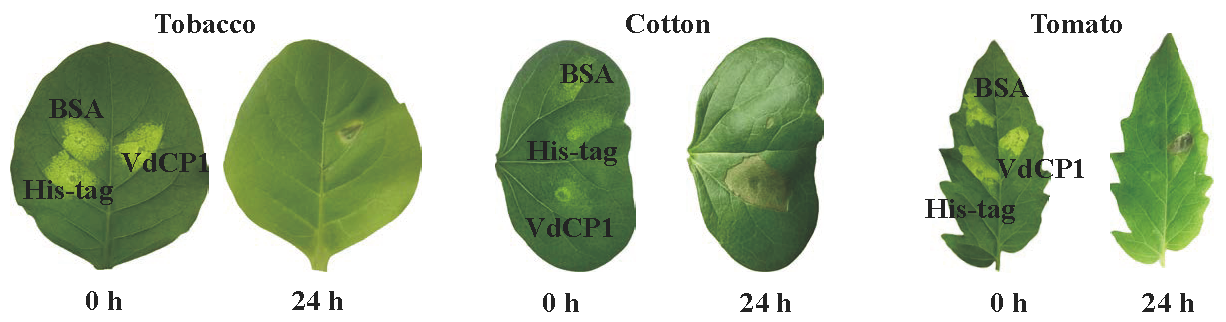

Supplement: Figure S5 — Assay of the HR-inducing ability of VdCP1. Tobacco, cotton and tomato leaves were infiltrated with 200 μM of VdCP1, and photographs were also taken with front illumination at 24 h post infiltration to demonstrate the effect on the leaves. His-tag and BSA (at the same concentration of VdCP1) were controls. [file Image5.TIFF]

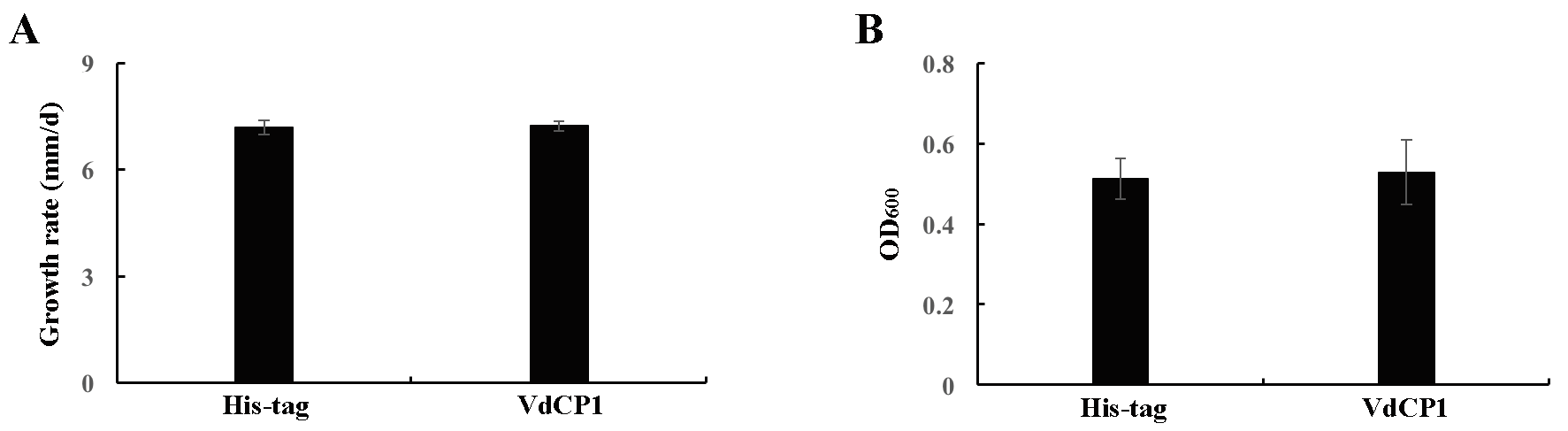

Supplement: Figure S6 — The direct toxic activity of VdCP1 on pathogens. (A) At 5 days after the inoculation of B. cinerea on PDA plates containing 100 μM VdCP1 or His-tag, the growth rate of B. cinerea on PDA plates was calculated by measuring the diameter. (B) At 2 days after the inoculation of P. syringae pv. tabaci in KB Rif100 liquid medium containing 100 μM VdCP1 or His-tag, the growth of P. syringae pv. tabaci was calculated based on the OD600. [file Image6.TIFF]

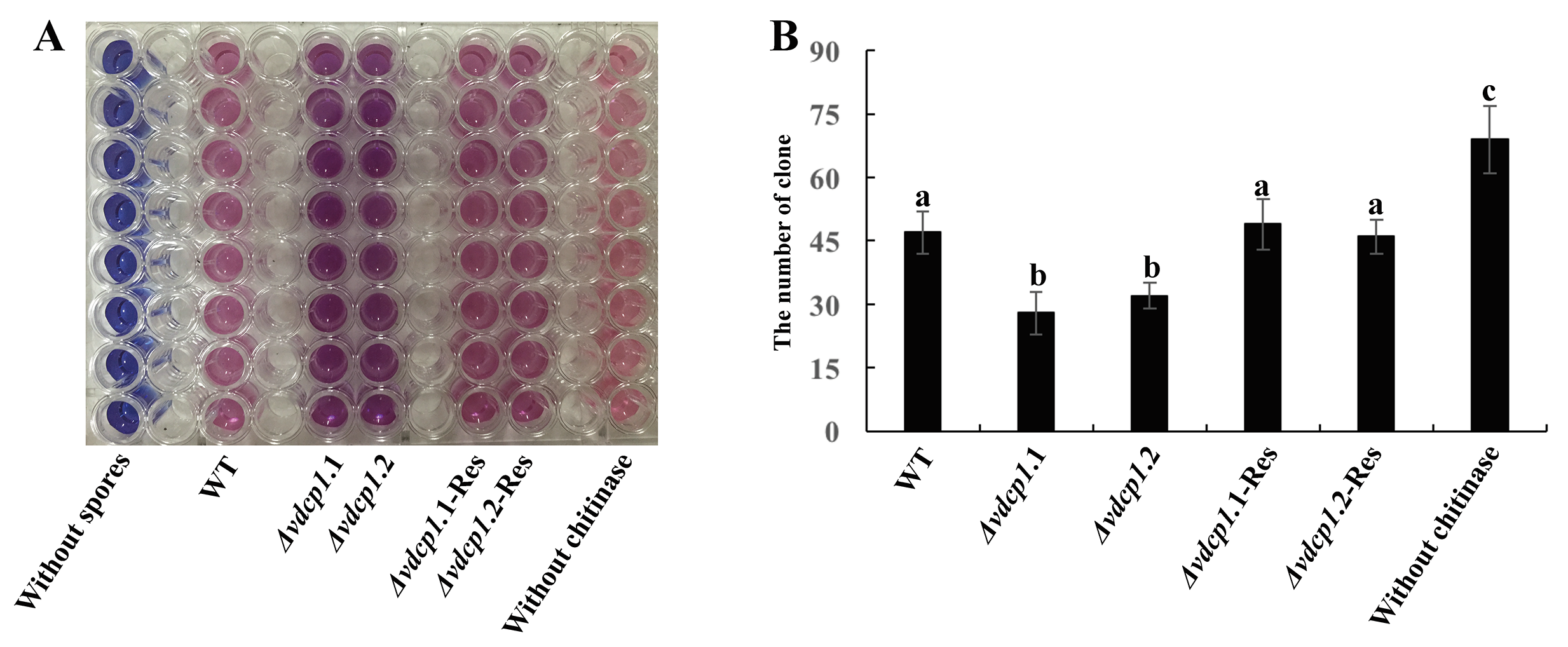

Supplement: Figure S7 — Color changes in PDB and the number of surviving spores. (A) Effect of the fungal spores on the color of PDB containing resazurin. PDB containing resazurin and chitinase without spores was blue, and PDB containing resazurin and chitinase with spores changed to pink, the color of the PDB with Δvdcp1 spores was deeper than that of the PDB with WT and Δvdcp1-Res spores. PDB containing resazurin and spores without chitinase was light pink. (B) The PDB diluted appropriately in media was spread on PDA plates, and then the number of clones was calculated to show the spore survival. Letters above the bars indicate a statistically significant difference (n = 3, p < 0.05 by Tukey-Kramer's test). [file Image7.TIFF]
