# Supplementary material for: The Verticillium dahliae SnodProt1-Like Protein VdCP1 Contributes to Virulence and Triggers the Plant Immune System
Source: Front Plant Sci. 2017 Oct 31;8:1880. doi: 10.3389/fpls.2017.01880 (PMC5671667; doi:10.3389/fpls.2017.01880)
Supplement: Supplementary file 9 [file Table1.DOCX]

**Table S1.** Primers used in the study

| **Names** | **Primer sequence (5’-3’)** | **Description** |
| --- | --- | --- |
| HygF | TTGAAGGAGCATTTTTGGGC |  |
| HygR | TTATCTTTGCGAACCCAGGG | 1800-bp hygromycin resistance cassette |
| vdcp1aF | TGCTCCGTTCCAACCTTCTAGT |  |
| vdcp1aR | GCCCAAAAATGCTCCTTCAAGTTGGAAGTTGAGGGGTGATT | 1000-bp 5’ region of *vdcp1* |
| vdcp1bF | CCCTGGGTTCGCAAAGATAAGGCGTCTGCGTTATTAGAGGG |  |
| vdcp1bR | AAGAACGGATCTATGACATGA | 1000-bp 3’ region of *vdcp1* |
| NestF | GGGGACAAGTTTGTACAAAAAAGCAGGCTTGCTCCGTTCCAACCTTCTAGT |  |
| NestR | GGGGACCACTTTGTACAAGAAAGCTGGGTAAGAACGGATCTATGACATGA | Construct *vdcp1* deletion mutant |
| vdcp1CF | GGTACCAACGTGGGATCGCCGCTATCGTATC |  |
| vdcp1CR | TCTAGACGAGTAGGGCACTGACAGAGAGCAA | Complementary to *vdcp1* deletion mutant |
| Check1F | AATCACAACATTTGCTTTCAACACCA |  |
| Check1R | CCATGCAAACCCACTCCAATGCCAA | Check for knockout mutants |
| Check2F | ATGCAGCTGTCCAACCTCCTCG |  |
| Check2R  Check3F  Check3R | TCAGAGGCCGCACTTGCTCTTGT  Check1F  Check1R | Check for knockout mutants  Check for complementary strains |
| vdcp1F | CCGGAATTCGCCTCTGTCTCCTACGACAA |  |
| vdcp1R | GCTCTAGACCGAGGCCGCACTTGCTCTTGT | *vdcp1* amplification |
| NtEF1αF | AGCCAAACCCTAGCTCCATT |  |
| NtEF1αR | GATGAAGATACTCACAGAAAGA | *Nicotiana tabacum* actin gene (qPCR) |
| NtPR1aF | CGTTGAGATGTGGGTCGATG |  |
| NtPR1aR | CCTAGCACATCCAACACGAA | *Nicotiana tabacum* pathogenesis-related 1 (qPCR) |
| NtPR5F | CTCATGCTGCCACTTTTGAC |  |
| NtPR5R | CTCCAAGATTGGCCTGAGTC | *Nicotiana tabacum* pathogenesis-related 5 (qPCR) |
| NtHIN1F | CTGCAACCCATGTAGCTGTC |  |
| NtHIN1R | GGTCGAAGAACGAGCCATAG | *Nicotiana tabacum* HR marker gene (qPCR) |
| NtHSR203JF | TGCCGTCAAAGATGTAGTCG |  |
| NtHSR203JR | CAGCATGGCTGACACAAAAG | *Nicotiana tabacum* HR marker gene (qPCR) |
| NtLOXF | CTTTAAGAGGAGATGGAACT |  |
| NtLOXR | TCTAAGCTCATAAGCAATGG | *Nicotiana tabacum* lipoxygenase (qPCR) |
| NtEDS1F | GGAGAATGGGAGAAGCAGAA |  |
| NtEDS1R | GAACGCATCATAATACCCGA | *Nicotiana tabacum* enhanced disease susceptibility 1 (qPCR) |
| vdActinF | GACAATGGTTCGGGTATG |  |
| vdActinR | GACAATGGAAGGGAAGAC | *Verticillium dahliae* actin gene (qPCR) |
| qvdcp1F | AGCAGGTCAAGAAGTTCC |  |
| qvdcp1R | GATGGCGAGGACATTGAT | *vdcp1* (qPCR) |
| GhActinF | CTCTTCCAGCCATCTCTTAT |  |
| GhActinR | CACTGAGCACAATGTTACC | *Gossypium hirsutum* actin gene (qPCR) |
| GLUF | CATTGATATGACCTTGATCG |  |
| GLUR | GTGAGATATCCCTTGGATTG | *Gossypium hirsutum* pathogen-induced glucanase (qPCR) |
| CHTF | ACCAAGCTACTCGCAAGAGG |  |
| CHTR | CGGAAGCGCAGTAAGATGA | *Gossypium hirsutum* pathogen-induced class I chitinase (qPCR) |
| GhNPR1F | TCTCGAAAATAGAGTTGG |  |
| GhNPR1R | CCTCTTGAATCCTGAAAG | *Gossypium hirsutum* NPR1 (qPCR) |
